# Symbiotic bacteria and fungi proliferate in diapause and may enhance overwintering survival in a solitary bee

**DOI:** 10.1093/ismejo/wrae089

**Published:** 2024-05-20

**Authors:** Shawn M Christensen, Sriram N Srinivas, Quinn S McFrederick, Bryan N Danforth, Stephen L Buchmann, Rachel L Vannette

**Affiliations:** Department of Entomology and Nematology, University of California Davis, Davis, CA 95616, United States; Department of Entomology and Nematology, University of California Davis, Davis, CA 95616, United States; Department of Entomology, University of California Riverside, Riverside, CA 92521, United States; Department of Entomology, Cornell University, Ithaca, NY 14853, United States; Department of Ecology and Evolutionary Biology, The University of Arizona, Tucson, AZ 85719, United States; Department of Entomology and Nematology, University of California Davis, Davis, CA 95616, United States

**Keywords:** core microbiome, host–microbe, solitary bee, insect development

## Abstract

Host–microbe interactions underlie the development and fitness of many macroorganisms, including bees. Whereas many social bees benefit from vertically transmitted gut bacteria, current data suggests that solitary bees, which comprise the vast majority of species diversity within bees, lack a highly specialized gut microbiome. Here, we examine the composition and abundance of bacteria and fungi throughout the complete life cycle of the ground-nesting solitary bee *Anthophora bomboides standfordiana*. In contrast to expectations, immature bee stages maintain a distinct core microbiome consisting of Actinobacterial genera (*Streptomyces*, *Nocardiodes*) and the fungus *Moniliella spathulata*. Dormant (diapausing) larval bees hosted the most abundant and distinctive bacteria and fungi, attaining 33 and 52 times their initial copy number, respectively. We tested two adaptive hypotheses regarding microbial functions for diapausing bees. First, using isolated bacteria and fungi, we found that *Streptomyces* from brood cells inhibited the growth of multiple pathogenic filamentous fungi, suggesting a role in pathogen protection during overwintering, when bees face high pathogen pressure. Second, sugar alcohol composition changed in tandem with major changes in fungal abundance, suggesting links with bee cold tolerance or overwintering biology. We find that *A. bomboides* hosts a conserved core microbiome that may provide key fitness advantages through larval development and diapause, which raises the question of how this microbiome is maintained and faithfully transmitted between generations. Our results suggest that focus on microbiomes of mature or active insect developmental stages may overlook stage-specific symbionts and microbial fitness contributions during host dormancy.

## Introduction

The ecological and evolutionary success of a wide range of insect species has hinged on partnerships with microbes [1, 2]. Bacterial and fungal metabolism can facilitate novel resource use for insect hosts via synthesis of limiting nutrients [3], evasion or detoxification of diet defenses [4], digestion of recalcitrant substrates [5], or serving as food themselves [6]. Bacteria and fungi may also provide insect hosts defense from predation [7, 8], pathogens [9, 10], or food spoilage [11], yet the insect host breadth and life cycle dynamics of such interactions remain poorly understood.

Most specialized bee–microbe interactions are described in adult corbiculate social bees [12–14]. Their microbiomes function in digestion of pollen, regulation of immunity, and suppression of pathogen growth [15–17]. The vast majority of bee species, however, are solitary, lacking cooperative brood care, foraging, and feeding [18]. Unlike social bees, solitary bee species studied to date generally have less specific and less consistent microbiomes [19, 20].

A solitary bee spends up to 80% of its life developing inside a sealed brood cell [18]; during this time, the developing bee undergoes significant changes in metabolism and morphology—most profoundly during diapause and metamorphosis, respectively [21–24]. Brood cells are created and provisioned with pollen and nectar by the adult female: in some cases, provisions can be dominated by lactobacilli; however, these bacteria do not persist through development [25–27]. Annual life cycles, solitary nesting, complete metamorphosis, and lack of direct brood care are hypothesized to impede the development of a specialized core microbiome in solitary bees [13, 23]; instead, they are thought to acquire and filter microbes from the environment anew each generation, excepting occasional endosymbiotic bacteria [28], resulting in variable microbial communities among individuals and populations.

Here, we characterize the composition, abundance, and potential functions of the bacterial and fungal microbiome of the solitary bee *Anthophora bomboides stanfordiana* Kirby, 1837 (Hymenoptera: Apidae) over eight developmental stages, from two geographic sites, over 2 years using amplicon sequencing, qPCR, microbial isolations, and *in vitro* trials and assays. We describe a uniquely consistent core microbiome throughout brood development of this solitary bee species and test adaptive hypotheses regarding microbial effects on bee ecology and metabolism.

### Study system

**Figure 1:**
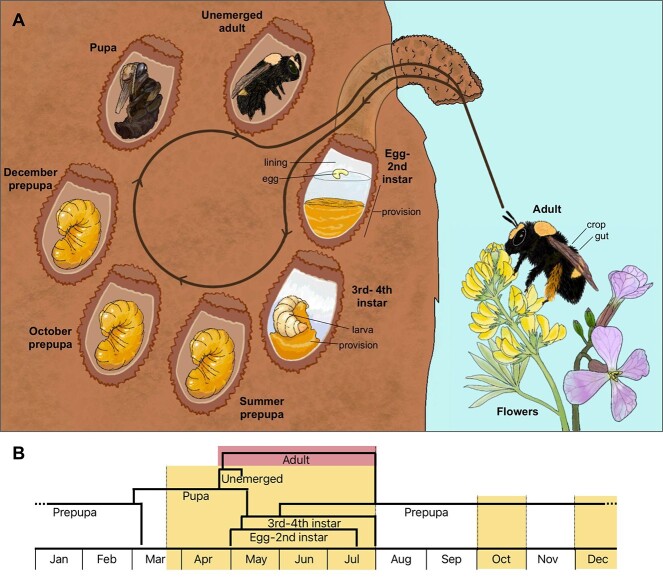
Life cycle of *A. bomboides* indicating sampled stages. (A) Full circle represents one year, with labeled brood cells illustrating stages that were sampled. The egg-second instar stage includes pollen provisions that contained liquid nectar, only provision was sampled. The third to fourth instar stage contained pollen, but no nectar and was separated into larva and provision samples, summer prepupae (collected pre-August) had recently eaten the cell lining, defecated, and turned yellow-orange. Overwintering prepupae collected in October and December are categorized as such. Pupal and unemerged adult stages were collected in spring; the latter were distinguished by complete development of hairs. Active foraging adults were collected and dissected for crop and gut samples. Stages are listed stepwise as they occur for one bee. Black text and lines indicate which parts of the stage were used for further analysis, excepting “lining” and “egg,” which are labeled for illustrative purposes. Absence of lines indicate that the whole bee was used as the sample for that stage. (B) At the population level, some stages overlap in time. Highlighted sections indicate months when sampling occurred. Yellow (lighter shading) indicated that sampled stages occur within the brood cell; red (darker shading) indicates that sampled stage occurs outside of the brood cell. Illustration by S. Christensen.

*Anthophora bomboides stanfordiana* (from here: *A. bomboides)* is a gregariously nesting solitary bee, inhabiting bluffs along the western coast of North America. *Anthophora bomboides* is a generalist forager (polylectic), but at our sites, prefers nectar from radish (*Raphanus sativus*) and pollen from lupine (*Lupinus arboreus*) (Fig. 1, other forage noted in Supplemental Methods). An adult nest in densely populated sites (nests within centimeters, not connected) contains tens to over a hundred thousand bees [29]. Nests are dug using fresh water from a nearby seep or creek to soften the hard dirt (Fig. 1A) [30]. Once a brood cell is excavated, the inside is lined with a thick waxy secretion. This lining has a cheesy aroma and has been studied in closely related *A. abrupta;* it is produced in the female’s hypertrophied Dufour’s gland and consists mostly of triglycerides that are converted to solid diglycerides during cell construction [31]. The lining is eaten by the larva as food after the pollen provision is consumed and just before diapause as a prepupa (Fig. 1A), and is thought to be highly specialized for consumption [31, 32].

The brood provision is initially very liquid, containing ~630 μl of nectar (Fig. 1) [30]. After hatching from the egg, larvae (first to second instars) consume the nectar, then pollen (third to fourth instars), and finally the cell lining; the white larva becomes yellow prepupa, defecates, and diapauses from fall to early spring (Fig. 1). In early spring, the prepupae exit diapause and pupate; adults emerge in late spring (Fig. 1) [30].

Two nest sites on the California coast were sampled in this study: McClure’s Beach (Point Reyes National Seashore, Marin County, CA, USA) and Bodega Head (Bodega Bay Marine Lab, Sonoma County, CA, USA). The sites are 9.8 miles apart and separated by a 5-mile open stretch of ocean. Both nesting sites have been active for at least four decades [30]. Because of the largely Mediterranean climate along the coast, the nesting period is warm and quite dry, but winters are wet and cool.

## Materials and methods

### See SI Methods for further details

#### Sample collection

Sampling was carried out from 2021 to 2023 at Point Reyes National Seashore and Bodega Head, both in CA, USA. *Anthophora bomboides* females and brood cells were collected using nets and soil tools, respectively. Brood cell contents, as well as adult GI tracts, were separated by careful dissection with sterilized instruments. Additionally, nearby flowers, water used for nest construction, and soil from the nesting area were collected to assess environmental microbes.

#### DNA extraction

Samples were added whole to DNA extraction, following preprocessing. Extraction for all samples was done per manufacturer’s instructions with the DNeasy PowerSoil Pro kit; four blanks were included (SI Methods Tables 1 and 2).

#### Amplicon sequencing

Amplicon sequencing of extracted DNA was done to assess bacterial and fungal community composition using the 16S rRNA gene and ITS region on MiSeq (Illumina) [33]. For bacteria, primers 799F/1115R amplifying the V5/V6 region of the 16S rRNA gene were used to limit mitochondria and chloroplast amplification (799F = 5'-AACMGGATTAGATACCCKG-3′/ 1115R = 5'-AGGGTTGCGCTCGTTG-3′) [34, 35]. For fungi, primers ITS1F/ITS2 were used (ITS1F = 5'-CTTGGTCATTTAGAGGAAGTAA-3′/ ITS2 = 5'-GCTGCGTTCTTCATCGATGC-3′) to amplify the variable length ITS1/2 region. Sequences were analyzed in R (4.1.1) [36] with primarily the DADA2 package (1.22.0) [37], phyloseq (1.38.0) [38], vegan (2.6.4) [39], microbiome (1.23.1) [40], and ggplot2 (3.4.2) [41]. See supplemental code for further details.

##### Bacteria

Reads were filtered and trimmed, followed by error rate calculation, dereplication, denoising, merging, and chimera removal; see supplemental code (Bacteria, code1) and data (“16S_track_reads”). Amplicon sequence variants (ASVs) were inferred via DADA2 (1.22.0), and then taxonomy was assigned using the Silva ​​138.1N99 database for bacteria [42]. Mitochondria and chloroplast assigned reads were removed, as were contaminants via the Decontam package (1.14.0) [43]. Samples with <300 reads were then removed from further analysis, leaving *n* = 86 samples. As both soil samples were removed at this step, we separated and reanalyzed these without filtering to determine general composition (Fig. S1).

##### Fungi

Primers were removed using Cutadapt [44]. Reads were filtered and trimmed, then error rates, dereplication, denoising, merging, and chimera removal were done with default parameters; see supplemental code (Fungi, code1) and data (“ITS_track_reads”). ASVs were inferred via DADA2 (1.22.0) and taxonomy assigned using UNITE general release dynamic database (29.11.2022) [45]. Nonfungal assigned reads were removed, and the Decontam package (1.14.0) [46] was used to remove contaminants. Samples with fewer than 300 reads were removed from further analysis, leaving *n* = 93 samples.

#### Statistical analysis

Community differences between inside and outside of brood cell, as well as for stage-specific community separation, were evaluated by PERMANOVA based on Bray–Curtis (BC) distances for both bacteria and fungi. The relative abundance of both Actinobacteria and *Moniliella* inside versus outside of brood cell were compared between the groups with Kruskal–Wallis test.

For core taxa detection, we used the “microbiome” package “plot_core” function to visualize a wide range of prevalence (0%–100%) and abundance (0.01%–20%) thresholds for both bacteria and fungi in the style of a heatmap for clarity and to allow for nuance in interpretation of what may be considered core taxa.

Differences in relative abundance of *Streptomyces* was evaluated with Kruskal–Wallis [36] followed by Dunn’s test for multiple comparisons [47] with Bonferroni *P* value correction [48]. To determine “actual” abundance, we combined the qPCR data with the amplicon data by multiplying the total bacterial copy number by the proportion assigned to *Streptomyces* in each sample*.*

#### qPCR

Bacterial copy number was quantified with standard DNA intercalating dye (SYBR) based qPCR on the same extracted samples that were sent for amplicon sequencing. Identical primers (799F/1115R) were used. Reactions were performed in triplicate for each sample, blanks and standards were included in each plate, and a Cq cutoff for blanks was established at 31.

To translate Cq values to copy number, we purchased a plasmid containing the relevant sequence from *Nocardiodes luteus* (ASV_5) at a known concentration. This was used to create a standard curve (*R*^2^ = 0.98) that was then used to convert Cq values to log(copy number). Using amplicon data, we also adjusted the final qPCR copy number to remove the proportion of reads in each sample that had been assigned to mitochondria (no chloroplast reads were assigned). Flower and water samples had to be concentrated to ensure sufficient DNA for sequencing during pre-processing, and thus, bacteria in these samples could not be reliably quantified with qPCR.

Fungal copy number was quantified with probe-based (Taq-Man) qPCR using a previously established system, FungiQuant [49] on the 18S rRNA gene primers FungiQuant-F = 5′-GGRAAACTCACCAGGTCCAG-3′ and FungiQuant-R = 5′-GSWCTATCCCCAKCACGA-3′, along with the fluorescent probe FungiQuant-Prb = (6FAM) 5′-TGGTGCATGGCCGTT-3′ (MGBNFQ). Reactions were performed in triplicate for each sample. Blanks and standards were included in each plate. We purchased a plasmid containing the relevant sequence from *Moniliella oedocephalis* (#NG_062174) at a known concentration. This was used to create a standard curve (*R*^2^ = 0.98) that was used to convert Cq values to log(copy number). To evaluate differences in copy number between stages for both bacteria and fungi, we used Kruskal–Wallis tests followed by Dunn’s test for multiple comparison with BH *P* value correction [50].

#### Inhibition

Strains were isolated by plating brood cell contents on Tryptic Soy Agar (TSA) or Yeast Media Agar (YM) and identified based on Sanger sequencing followed by NCBI BLAST [51]. Bacterial identification used the 16S rRNA gene and fungal identification was based on ITS or 18S rRNA gene D1/D2 region (SI Methods Table 3). For all inhibition trials, we used Tryptic Soy Agar (TSA) without any antimicrobials. *Streptomyces* strains were inoculated, and 10 days later, fungal plugs were added to the center (five replicate plates per combination). After 7 days, fungal radius measurements were taken. These were analyzed via Kruskal–Wallis tests followed by Dunn’s test for multiple comparison with Bonferroni *P* value correction [48].

#### Sugar and sugar alcohols

Samples of whole larvae, prepupae, and pupae, as well as one pollen provision from a fourth instar larva, were extracted for sugar and sugar alcohol (SSA) analysis, see SI Methods. Separation of sugars was performed on Thermo UltiMate 3000 HPLC system via modified Waters Application Note: WA60110. Each sample was run twice, standards of erythritol, sorbitol, fructose, glucose, sucrose, xylose, maltose, glycerol, and trehalose were run two to five times. Peak area was calculated by the Thermo Fisher Chromeleon software. To identify differences in sample groups based on SSA profiles, we used Principal Components Analysis (PCA) after normalization by Hellinger transformation. After calculation of the BC distance matrix, PERMANOVA and pairwise PERMANOVA were used to determine differences in composition of SSA by sample group, *P* value correction by FDR [50].

## Results

### *A. bomboides* brood cell microbial communities are distinct from the environment and dominated by Actinobacteria and the fungus *Moniliella spathulata*

**Figure 2:**
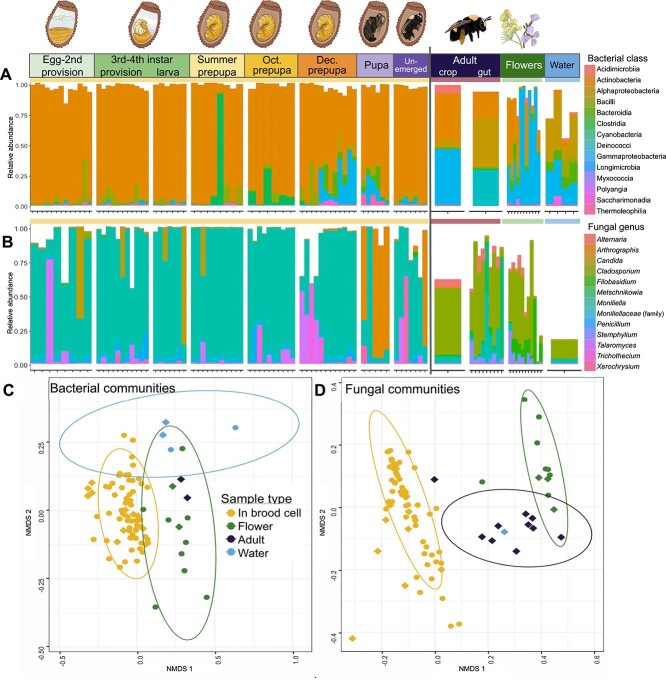
*Anthophora bomboides* brood cell samples are dominated by Actinobacteria and *Moniliella,* differing significantly from adults and environment. (**A, B**) Stages arranged in order of development, followed by environmental samples (flowers and water). Dirt samples yielded no sequences after quality control and filtering, see Fig. S1. Yellow horizontal bar indicates stages/samples which occur within the brood cell, red indicates adults collected outside of the brood cell, green indicates flower samples, and blue indicates water samples where adult bees collect water for nest construction. Each vertical column represents one sample, fill corresponds to proportion of sample reads belonging to each taxa (colors in key). Black vertical line separates inside (left) versus outside (right) of the brood cell. The same samples were sequenced for both bacteria and fungi, but discrepancies occur when a sample was filtered out due to low read count in one sequencing run, but not the other, such as the adult gut sequencing well for fungi and poorly for bacteria. (**A**) Top 500 bacterial ASVs, colored by class, via sequencing of V5/V6 region of 16S rRNA gene. Actinobacteria (orange) dominate within the brood cell. Kruskal–Wallis χ^2^ = 37.6, df = 1, *P* value = 8.7e-10 comparing relative abundance of Actinobacteria from inside brood cell samples (mean 84.9%; yellow horizontal bar; left of vertical black line) to outside brood cell samples (mean 20.1%; red, green, blue bars; right of line). *n* = 86 samples. (**B**) Top 15 fungal ASVs, via sequencing of ITS region, colored by genus. *Moniliella* (light blue) dominate within the brood cell. Kruskal–Wallis χ^2^ = 43.9, df = 1, *P* value = 3.3e-11 comparing relative abundance of *M. spathulata* from inside brood cell samples (mean 72.3%; yellow bar; left of line) to outside brood cell samples (mean 5.4%; red, green, blue bars; right of line). *n* = 93 samples. (**C, D**) For both plots, color indicates sample type, shape indicates sample site. Yellow corresponds to samples from inside the brood cell, green corresponds to flower samples, purple to adults that were free-flying outside of the brood cell, and blue to water samples. Circles were sampled from McClure’s beach site, diamonds from Bodega Head site. (**C) **Nonmetric multidimensional scaling (NMDS) of weighted BC distance for bacterial communities (stress = 0.19). PERMANOVA with sample type as a predictor *R*^2^ = 0.07, *F* = 2.2, *P* value <0.001. (**D**) NMDS of weighted BC distance for fungal communities (stress = 0.1). PERMANOVA with sample type as a predictor *R*^2^ = 0.25, *F* = 10.12, *P* value <0.001.

For both bacteria and fungi, brood cell microbial composition was distinct from environmental sources and from adult gut samples (Fig. 2C and D; Fig. S2, SI Methods Table 1). Actinobacteria were predominant during all stages of brood cell development in significantly higher relative abundance compared to samples from outside of the brood cell (K–W *χ*^2^ = 37.6, df = 1, *P* value = 8.7e-10; Fig. 2A). All sample groups contained Actinobacteria, but brood cell samples had 84.9% (*n* = 69, SD = 17.9) relative abundance, adult bee samples had 29.9% relative abundance (*n* = 2, SD = 12.7), and environmental samples had 18.8% relative abundance (*n* = 15, SD = 20.4).

To determine if Actinobacteria are specifically affiliated with *A. bomboides* we compared relative abundance of the Actinobacteria-assigned ASVs (489 total) in brood cell and environmental samples (Fig. S3). With a 0.1% detection threshold, 60% were found exclusively in the brood cell samples (294 ASVs), 17% were found in both brood cell and environmental samples (64 ASVs), and 25% were only detected in environmental samples (123 ASVs). Of the 64 ASVs shared between brood and environmental samples, 54% were found in only one environmental sample, mostly flower samples likely visited by foraging *A. bomboides* (*Raphanus sativus, Erigeron glaucus*). These 64 shared ASVs make up on average 9% of the reads in environmental samples (SD = 13.56). Six Actinobacteria ASVs are shared between the adult bee gut samples and the brood cells, comprising an average of only 1.6% of the reads within the brood cell samples (SD = 1.85).

Fungal communities within brood cells were dominated by *Moniliella spathulata* across all stages except the pupal stage (Fig. 2B). *Moniliella spathulata* was detected in every brood cell where it comprised on average 72.3% of sequences (*n* = 71, SD = 29.6), significantly greater than in adult bees or environmental sources (Fig. 2B, K–W *χ*^2^ = 43.9, df = 1, *P* value = 3.3e-11). *Moniliella spathulata* was detected in all but one adult GI tract sample, where its average relative abundance was only 10.9% (*n* = 10, SD = 16.1). Three environmental samples (*R. sativus* bulked 15 flowers, *Carpobrotus edulis* flower, water sample) contained *M. spathulata;* the mean relative abundance in all environmental samples was 0.7%​​ (*n* = 12, SD = 1.57).

### *A. bomboides* brood cells host a core microbiome composed of select Actinomycete genera and *M. spathulata*

**Figure 3:**
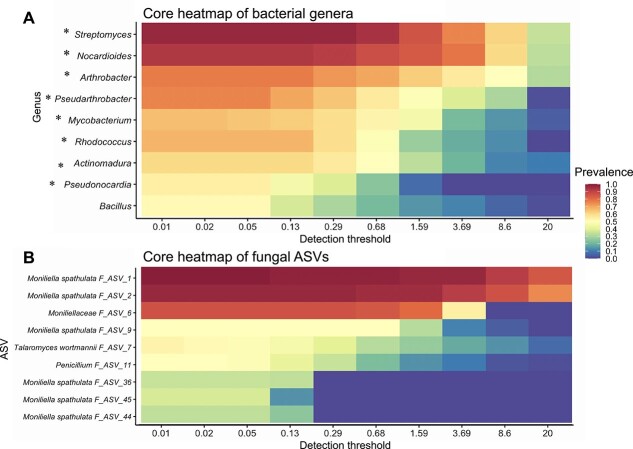
*Anthophora bomboides* brood cells host a core microbiome composed of actinomycetes and *Moniliella*. Core microbiome heat maps for bacterial genera (**A**) and fungal ASVs (**B**), indicating prevalence at increasing detection thresholds. Prevalence is the proportion of samples containing the indicated taxa; detection threshold is the minimum relative abundance that needs to be present in a sample for it to be counted. Together, these separate core taxa (high prevalence and abundance) from other taxa. Top taxa arranged in decreasing order down the *y* axis, prevalence for each taxa is indicated by color, with 1 (dark red) meaning that the taxa is present in all samples, and 0 (dark blue) indicating it is present in none of the samples at each detection threshold (*x* axis, % of reads). (**A**) All bacterial genera with an asterisk (*) belong to Actinobacterial class. (**B**) Fungal ASVs labeled at the most specific assigned taxonomic level, see Fig. S5 for relative abundance of fungal ASVs 1 & 2.

We next evaluated the presence and composition of a core microbiome in *A. bomboides* brood cells. Bacterial core was defined at the genus level for samples inside the brood cell, because the genera seemed to remain quite consistent despite diversity at the ASV level. At a prevalence of 65% and detection threshold of 0.1%, six genera (*Streptomyces, Arthrobacter, Nocardioides, Mycobacterium, Pseudarthrobacter,* and *Rhodococcus*) comprise the bacterial core. At the stricter prevalence cutoff of 90%, *Streptomyces* and *Nocardioides* remain core genera (Fig. 3A). Regardless of the specific numerical cutoff, the top eight genera that could constitute the core are all Actinobacteria (marked with * on Fig. 3A).

The fungal core microbiome was defined at the ASV level for samples inside the brood cell. Two ASVs in particular, ASV_1 and ASV_2, both assigned to *M. spathulata,* are present in 97% of samples at a detection threshold of 0.5% and in 88% of samples at a detection threshold of 5% (Fig. 3B). ASV_6, also belonging to *Moniliellaceae*, could be considered core at lower thresholds, but no other ASVs approach inclusion in the fungal core within the brood cell (Fig. 3B).

### Microbial abundance peaks in during bee diapause

**Figure 4:**
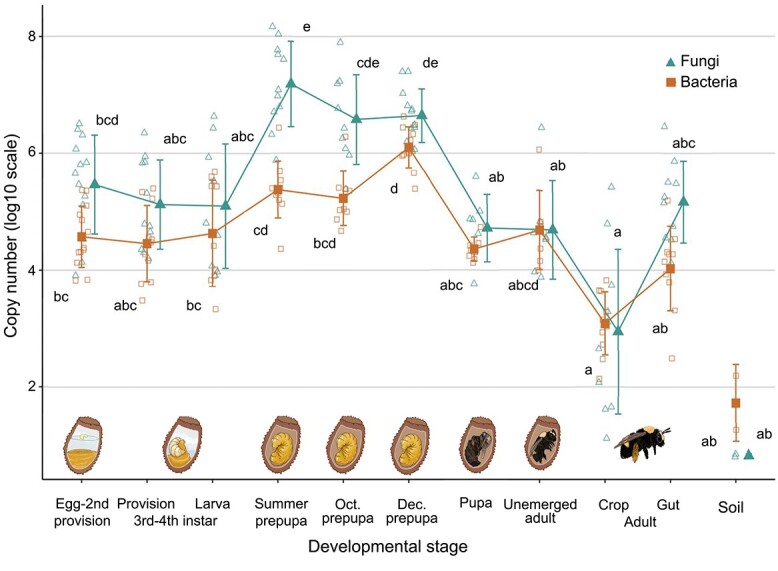
*Anthophora bomboides* microbial copy number is highest in diapausing prepupae. Filled points represent the mean of the log10(copy number) by developmental stage, error bars +/−1 SD log(copy number) of each developmental stage for both bacteria (orange squares) and fungi (teal triangles). Smaller open points represent individual samples. Bacterial copy number has been adjusted to remove nonbacterial reads, as determined by amplicon sequencing with identical primers. Kruskal–Wallis test indicates significant differences in bacterial and fungal copy number based on bee developmental stage, (bacteria—K–W χ^2^ = 66.7, df = 10, *P* value = 1.9e-10; fungi—K–W χ^2^ = 67.0, df = 10, *P* value = 1.6e-10). Lettering indicates differences via Dunn’s multiple comparisons; above for fungi and below for bacteria (P value < 0.01).

To quantify abundance of bacteria and fungi throughout the bee life cycle, we conducted qPCR using the same bee samples as above. Bacterial copy number increased through bee development (Fig. 4, K–W χ^2^ = 66.7, df = 10, *P* value = 1.9e-10). Specifically, the bacterial copy number increases through larval development, peaking during December, mid-diapause, and decreasing after pupation; the bacterial copy number in December prepupae is 33 times higher than in egg-second instar (1.3e6, 3.7e4).

Fungal qPCR was conducted using FungiQuant [49]. The fungal copy number also changed through development (Fig. 4, K–W χ^2^ = 67.0, df = 10, *P* value = 1.6e-10) increasing by a factor of 52 between egg-second instar stage and summer prepupae (2.8e5, 1.5e7), coinciding with consumption of the brood cell lining and defecation. Fungal copy number remained high through December before dropping by a factor of 83 between December prepupal stage and pupal stage (4.3e6, 5.2e4). Soil samples from each site were included; both showed very low density of fungi and bacteria.

### Stages within the brood cell have shifting bacterial and fungal communities, *Streptomyces* dominates in overwintering stages

**Figure 5:**
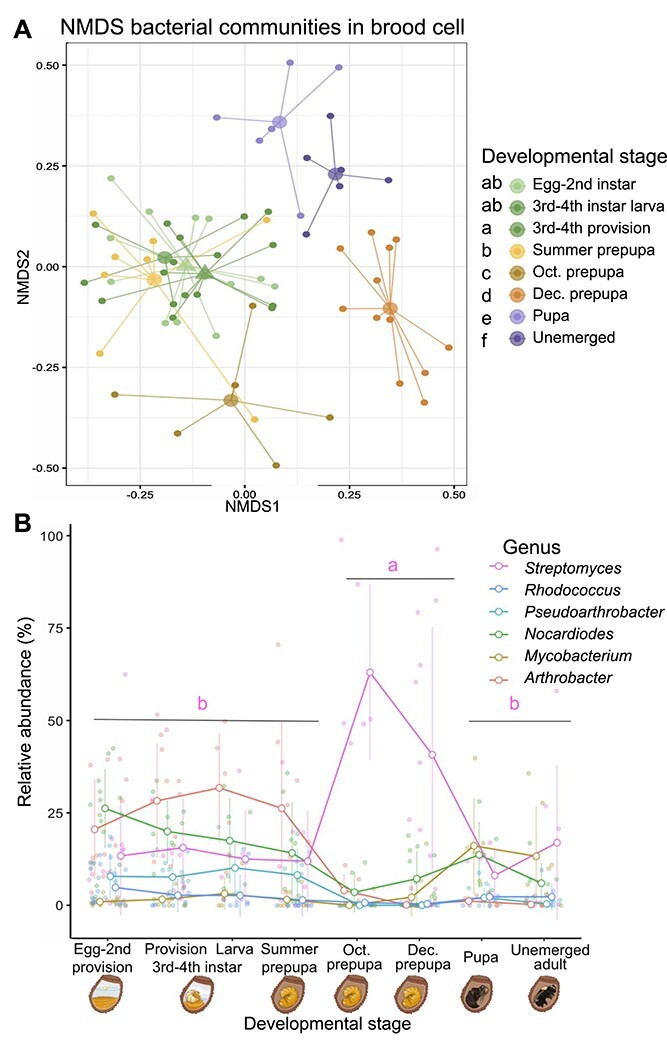
Bacterial communities shift with bee development, with increased *Streptomyces* abundance in overwintering stages. (**A**) NMDS of bacterial community BC distance with color indicating stage of brood cell development. Larger semitransparent dots indicate centroids, with lines from centroid to each point in the group. Triangular centroids indicate provision samples. Global PERMANOVA shows significant difference between stages (*R*^2^ = 0.25, *F* = 2.87, *P* value <0.001). Pairwise PERMANOVA of stages (FDR < 0.05) indicated with lettering on left side of figure key. (**B**) Relative abundance (% of total) of top six bacterial genera across bee developmental stages. Larger open circles represent mean relative abundance of the genus at the indicated developmental stage and are connected by lines. Error bars are (+/−1 SD). Each smaller shaded point represents the relative abundance of the corresponding genus in one sample. *Streptomyces* relative abundance varies through development (Kruskal–Wallis χ^2^ = 15.9, df = 2, *P* value = 0.0003) and is highest during overwintering (Oct.–Dec.) as compared to summer (egg-summer prepupa; Dunn’s test adj. *P* value <0.001), or spring stages (pupa-unemerged adult; Dunn’s test adj. *P* value <0.01).

We compared bacterial and fungal communities at different stages inside the brood cell to determine whether these communities shift at finer taxonomic scales and found that the community changed in consistent patterns through brood cell development (Fig. 5, Fig. S4). Microbial communities within the brood cell consistently contain a high proportion of Actinobacteria and *M. spathulata*, but the relative composition changed between stages after the summer prepupa stage (*P* value <.05, Fig. 5A). Fungal community composition was overall consistent between stages, but was distinct in summer prepupae and pupae (Fig. S5).

We further examined how the relative abundances of the top six bacterial core genera change with developmental stage. The most abundant genus, *Streptomyces*, peaks in abundance in overwintering prepupae, with average relative abundance of 48.5% (SD = 35.2, during October and December). *Streptomyces* relative abundance in the overwintering stages is significantly greater than summer (egg through summer prepupae, avg. 13.7% SD = 13.8, *P* value = 6e-4) and spring (pupae and unemerged adults, avg. 13%, SD = 16.1, *P* value = .003) stages by Dunn’s test (Fig. 5C).

### *Streptomyces* isolated from brood cells inhibits growth of filamentous fungi

**Figure 6:**
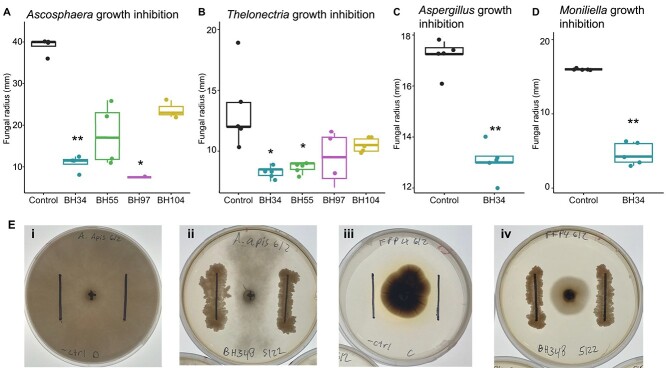
*Streptomyces* isolated from *A. bomboides* brood cells inhibit growth of filamentous fungi. Inhibition is shown by decrease in radius of fungi (mm, *y* axis) on plates when coinoculated with isolates of *Streptomyces* (BH104, BH34, BH55, BH97) (*x* axis, colors) from brood cells as compared to negative control (black). *Streptomyces* isolates plated 10 days prior to inoculation with a 6-mm diameter fungal plug. Comparisons to control by Dunn’s test, *P* values adjusted with Bonferroni (*P* value <0.05 = *, *P* value <0.01 = **). (**A–D**) Center lines correspond to the median, boxes define interquartile range (IQR), and whiskers extend +1.5 IQR. Note the difference in the scale of *y* axes. (**A**) Inhibition of *Ascosphaera apis,* a common pathogen of bee brood, on day 7 of coinoculation. Data represent 16 plates. BH34 and BH97 significantly reduced the radius of *A. apis.* Note that the 40-mm radius was the edge of the plate. Kruskal–Wallis χ^2^ = 12.3, df = 4, *P* value = 0.014. (**B**) Inhibition of *Thelonectria,* a potential pathogen isolated from an infected pupal cell, on day 7 of coinoculation. Data represents 24 plates. BH34 and BH55 significantly reduced the radius of *Thelonectria*. Kruskal–Wallis χ^2^ = 14.5, df = 4, *P* value = 0.005. (**C**) Inhibition of *Aspergillus flavus*, generalist bee pathogen, by BH34 on day 4 of coinoculation. Data represent 10 plates. Kruskal–Wallis χ^2^ = 6.9, df = 1, *P* value = 0.009. (**D**) Inhibition of *M. spathulata*, ubiquitous brood cell yeast, by BH34 on day 7 of coinoculation. Data represent 10 plates. BH34 significantly reduced the radius of *M. spathulata.* Kruskal–Wallis χ^2^ = 7.3, df = 1, *P* value = 0.007. I Plate images of fungi on day 7 of growth, when grown alone and with *Streptomyces* isolate BH034 showing decreased radius and qualitatively reduced hyphal density. (i) *A. apis* negative control, (ii) *A. apis* with *Streptomyces* isolate BH034, (iii) *Thelonectria* negative control, and (iv) *Thelonectria* with *Streptomyces* isolate BH034. Images were taken on a backlit LED screen to ensure identical lighting.

Fungal pathogens can thrive in the wet conditions of the overwintering period, which also coincided with peak abundance of *Streptomyces.* To test the hypothesis that brood-isolated *Streptomyces* inhibits growth of filamentous fungi, we used a plate-based coinoculation assay. Representative *Streptomyces* isolates from *A. bomboides* brood cells (BH34, BH55, BH97, BH104; Figs S6, S7, and S8) were tested against *Ascosphaera apis*, a devastating pathogen of bee brood, as well as *Thelonectria*, which we isolated from an infected pupa of *A. bomboides. Streptomyces* isolates from the brood cells were able to inhibit both pathogens. Although *Streptomyces* strains varied in their fungal growth suppression, *A. apis* was significantly inhibited by both BH34 (*P* value <.05) and BH97 (*P* value <.05) and *Thelonectria* was significantly inhibited by BH34 (*P* value <.01) and BH55 (*P* value <.05) on day 7 of coinoculation (Fig. 6A, B, and E). As BH34 was able to inhibit both pathogenic fungi, we then tested whether it would also inhibit *Aspergillus flavus*—another generalist bee pathogen, or *M. spathulata*—the core fungal taxa, using the same methods. We found that BH34 was also able to significantly inhibit the growth of both *Aspergillus flavus* (*P* value <.01, day 4) and brood cell–isolated *M. spathulata (P* value <.01, day 7; Fig. 6C and D).

### Sugar alcohol profiles distinguish developmental stages and coincide with changes in fungal abundance

**Figure 7:**
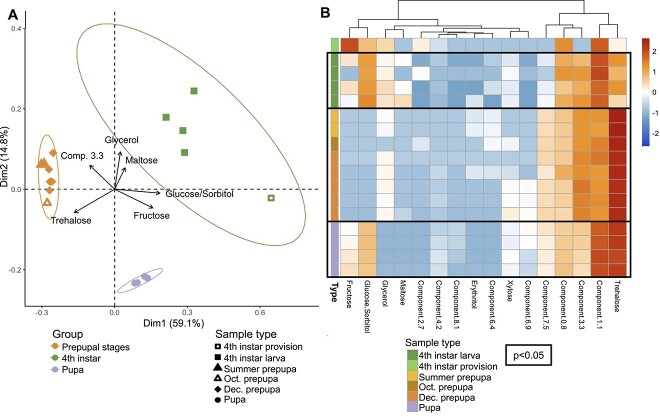
SSA composition differentiates bee developmental stages. (**A**) SSA composition of late larvae through pupal stages mapped by PCA, colored by stage. Ellipses indicate 95% confidence for sample groups. Axes labeled with % variance explained. Biplot of the six most influential SSA components; longer arrows indicate greater influence on sample separation, and direction indicates the alignment with the mapped PCs. Glucose and sorbitol had overlapping and thus indistinguishable peaks. (**B**) Composition of individual SSAs (columns) of each sample (rows). Samples grouped and colored by stage in the first column. SSA data were Hellinger-transformed and scaled to sample (relative abundances) with red indicating high relative abundance of that component (column) for that sample (row) and blue indicating low relative abundance within the sample. Black boxes indicate significant clustering of sample compositions *(P* value <0.05) via hierarchical clustering (multiscale bootstrap resampling).

The genus *Moniliella* is known for its industrial production of sugar alcohols and energy storing carbohydrates such as erythritol, glycerol, and trehalose [52, 53]. In insects, trehalose is protective against environmental stress, such as temperature extremes, dehydration, oxidation, and starvation [54, 55]. To determine whether *A. bomboides* stages exhibit changes in SSA composition in development that may coincide with proliferation of fungi, of which the vast majority are *Moniliella*, we analyzed fourth instar larvae (before fungal proliferation), prepupae (highest fungal abundance), and pupae (when fungal abundance drops, and *Moniliella* is no longer dominant; see Figs 2 and 4) to determine their composition of SSAs via high performance liquid chromatography with charged aerosol detection (HPLC-CAD). We found that the stages with high fungal abundance (prepupal, diapausing stages) have distinct SSA composition as compared to fourth instar and pupal stages (*P* value <0.01 by pairwise PERMANOVA; *P* values FDR-corrected, Fig. 7A). Specifically, glucose/sorbitol and fructose decline in relative abundance as bees develop from late-stage larvae to prepupae, while the disaccharide trehalose increases to high relative abundance in summer prepupae, coinciding with peak fungal abundance (*Moniliella*, Fig. 4). Trehalose remains the SSA with the highest relative abundance throughout diapause (Fig. 7B).

## Discussion

Our study provides a detailed characterization and demonstration of the potential for complex interplay of insect development with microbiome composition and abundance. In contrast to previously studied solitary bee species, we identified a complex core microbiome in the brood cells of the solitary bee *A. bomboides* that increases in abundance during diapause and persists through development; these findings are unique in several ways, which are discussed below.

### Stage-specific vulnerabilities

We find that *A. bomboides* hosts a distinct core microbiome of Actinobacteria and *M. spathulata* that increases in abundance and shifts in composition during diapause. Diapause is a programmed metabolic depression that allows an organism to wait out seasonal environmental conditions; it occurs in many organisms and is often a required stage before maturation [22]. Despite its importance for surviving these otherwise unsuitable conditions, insects are vulnerable to predation, freezing, drying out, and pathogen infection during diapause [7, 56–59]. Although the microbiome is known to mitigate impacts of environmental stressors on hosts [7, 14, 60, 61], the roles of microbial symbionts during diapause are poorly studied [62, 63]. Several studies show decreased microbial abundance and activity during diapause [64, 65], but other studies suggest its importance. In dormancy, the bacterial communities of animals change in composition and abundance [66–68]; can impact host gene expression [69], lipid accumulation, and survival [70], as well as provide pathogen suppression [9]; and are hypothesized to contribute to nutrient recycling systems while host systems are suppressed [71, 72].

The most common cause of brood mortality in *A. bomboides,* and solitary bees in general, is fungal infection during overwintering [58, 73]. Though diapausing insects retain innate immunity [74], it may be reduced or altered during this time [75, 76]. We found that bacteria in general, and *Streptomyces* specifically, attain the highest abundances during the winter months of diapause, with *Streptomyces* absolute abundance increasing by 46-fold between early provisions and December prepupae. This, combined with the demonstrated ability of brood-isolated *Streptomyces* to inhibit fungal pathogens of bees, suggests a defensive mutualism. *Streptomyces* are commonly found in soil, and many species and strains can produce antibiotics or antifungals; however, recent work has shown that insect-associated *Streptomyces* are more likely to inhibit pathogens than those found in soil, implying symbiont selection [77, 78]. This is also supported by the many examples of other insect-*Streptomyces* defensive mutualisms [79–82]. Alternatively, lowered bee immune defenses may allow *Streptomyces* to proliferate, or *Streptomyces* may be responding to season independently of the host; thus, further experiments are required to demonstrate the hypothesized mutualism. However, other bee species tend to exhibit low or undetectable microbial populations following defecation and in diapause, suggesting distinct biology in *A. bomboides* that supports symbiont growth.

Overwintering can lead to more subtle effects on survival via indirect chill injuries, which lead to a gradual failure to maintain homeostasis [22] and increased oxidative stress [83]. Many insects use antifreeze compounds, such as trehalose and glycerol, to reduce ice crystal formation and stabilize proteins [22]. *Moniliella,* the ubiquitous yeast-like fungus found with developing *A. bomboides*, are best known for their prolific production of sugar alcohols and trehalose [84]. By comparing pre-, during, and postdiapausing stages, we found that shifts in sugar alcohol composition corresponded to shifts in fungal abundance, with high levels of trehalose co-occurring with peak fungal abundance during diapause. Although insects are also capable of producing sugar alcohols and trehalose [85], and xerophilic yeasts may produce sugar alcohols to enhance their own survival in habitats with low water activity [86], other insect studies have shown microbiome correlation with [60], and direct contribution to, cold tolerance [69]. The temporal linkage of *M. spathulata* abundance and trehalose production suggests that symbionts could be involved in the production of compounds supporting host cold tolerance. As *M. spathulata* is also known to be oleaginous and able to accumulate lipids up to >60% of its dry weight, a role in lipid metabolism is also possible [87, 88]. Energy (lipid) storage for diapause can be reliant on microbial presence; recent work in mosquitoes showed that depleting microbial communities in prediapausing females prevents lipid accumulation, which rapidly and significantly lowers survival during diapause [70]. Broadly, it is possible that the developing bees are outsourcing some aspects of metabolism to their associated microbes while their own metabolism is suppressed during diapause [22]. These, as well as additional adaptive and nonadaptive hypotheses, will require further investigation.

### Transmission and acquisition

Previous work on solitary bee microbiomes indicates that shared flowers are the major mode of microbiome acquisition, resulting in variable microbial communities within species and between individuals with some exceptions of extreme filtering of environmental microbes resulting in dominance of lactic acid bacteria [20, 26, 27, 89–91]. In *A. bomboides,* however, brood microbiomes are distinct from the environmental samples, and patterns resemble vertical transmission, but this is not confirmed. Minimal bacterial ASV sharing between brood cells and flowers supports a distinct acquisition mode from that of other solitary bees (Fig. S3). Vertical transmission is especially likely for the fungal symbiont, *M. spathulata*, as nearly every brood cell from both sampled sites, even at the earliest stage (Fig. 2) contained the same two ASVs, indicating tight and effective control of transmission.

Though not generally found in flowers, Actinobacteria commonly inhabit soil environments. Our soil samples, however, were removed from the main sequencing dataset due to very low read count (but see Fig. S1), which was confirmed with qPCR (Fig. 4). We suspect that the low microbial densities may be due to high salt content (ocean spray) [92] and substrate composition, which is mostly decomposing granite (~73% sand), without visible roots or other organic material (see Supplemental Methods for full composition). It is possible that with more extensive substrate sampling, a link between soil and brood cell compositions could be found.

The core brood cell microbial composition was not consistent with adult GI tracts; 10 guts and 10 crops were sequenced—only one of each had enough bacterial reads to pass filtering, and moderately low bacterial density was confirmed by qPCR (Fig. 4). Fungal communities in the crop also had low density, whereas gut fungi were more abundant and more closely resembled environmental communities (both containined high *Cladosporium*), not brood cells (Fig. 2). This suggests a non-GI method of transmission. Another solitary hymenopteran, the beewolf (*Philanthus*), transfers its core brood associate (*Streptomyces*) to newly provisioned cells using specialized glands in the adult antennae [9]. Our results suggest a vertical mode of microbiome transmission in *A. bomboides,* but this hypothesis, along with the methods by which they maintain or select for this unique microbial composition, will require further study.

### Comparison to other insect microbiomes

The bacterial taxa that associate with *A. bomboides* have not been previously described as core associates of bees (Anthophila) (but see [93, 94]). The actinomycete genera *Streptomyces* and *Nocardioides* were present in over 90% of samples, and *M. spathulata* was found in nearly every cell as the dominant fungus. Other solitary species with specific bacterial associates commonly host environmentally acquired lactobacilli, or other floral and environmental microbes [20, 26, 89–91], whereas the social bee gut microbiome consists of a distinct set of bacterial genera not overlapping with the *A. bomboides* core [12, 13, 95]. A different *Moniliella* species (*M. megachilensis*) and *Streptomyces* have been isolated from brood of solitary *Megachile* bees [93, 96], which, like *A. bomboides,* are known to have hypertrophied triglyceride-producing Dufour’s glands [97, 98]. Though these taxa are rare in bees, they are in some ways comparable to the widely studied microbial associates of fungus-farming ants. Ant workers host actinomycetes (*Pseudonocardia, Streptomyces*) to protect the monoculture of cultivated fungus [99–101]. However, the data presented here show that although *Streptomyces* isolated from *A. bomboides* brood cells suppresses the growth of pathogenic fungi, they also suppress the growth of *M. spathulata*, the core fungal taxa (Fig. 6D). This observation suggests that microbial communities may exhibit temporal or small-scale physical niche partitioning between *Streptomyces* and *M. spathulata* that allows both to persist, or that conditions used in the plate assay result in different dynamics compared to the brood cell; this remains to be tested.

### Why *Anthophora*?

To date, *A. bomboides* is the only solitary bee that is now documented to associate with a complex and consistent core microbiome in the brood cell. What traits may support this specialized association? We hypothesize that the brood cell lining from the hypertrophied adult Dufour’s gland may be involved in maintaining this association. Some species within the genus *Anthophora* are unique in that their Dufour’s gland secretion has evolved from a thin waterproof cell lining to a thick, energy dense food source for developing brood [32]. This triglyceride-rich lining has been noted as being highly specialized, similar to royal jelly in honey bees or milk in mammals [31]. Adult females produce copious amounts of this secretion, using it to both line the cell and mix into the provision itself [31]. *Moniliella spathulata* is lipophilic and can degrade a wide range of over 150 hydrocarbons, perhaps the larvae’s consumption of the lipid lining just prior to diapause facilitates yeast proliferation in this stage [88]. Some Dufour’s secretions also have antibiotic properties, which, if present here, may also exhibit selective pressure in shaping the microbiome [102].

## Conclusions

We provide direct evidence of a consistent and abundant microbial community in developing *A. bomboides* solitary bees and antifungal activity of the abundant *Streptomyces,* which suggests a defensive symbiosis. Solitary bees are vulnerable to multiple sources of mortality during development, especially during overwintering. Increasing microbial titer during diapause, consistent composition across brood cells, and microbial phenotypes with clear links to bee life history suggest, but do not yet demonstrate, a mutualistic symbiosis. Specifically, two ASVs of the yeast *M. spathulata* were consistently found at both sampled sites and all developmental stages, with dramatic changes in density corresponding to significant shifts in sugar alcohol composition during overwintering, pointing to a role in cold tolerance. *Streptomyces* was found to be a potential defensive symbiont, inhibiting a variety of brood-pathogenic fungi, and dominating specifically the overwintering stages. These results highlight a few underappreciated aspects of insect-microbe symbiosis: (i) a complex and consistent microbiome can be maintained in the absence of sociality, (ii) bacteria and fungi may affect bee biology during diapause, and (iii) the mycobiome may be important and likely deserves additional study. Although much work remains to examine the ecology of this bee–microbiome symbiosis, our study reframes the conditions thought to maintain symbiosis and highlights novel research areas in exploring unique roles of the microbiome during host dormancy.

## Supplementary Material

Supplementary_Figures_Christensen_etal_2024_wrae089

SI_Methods_wrae089

## Data Availability

All data and code are publicly available through Dryad Data repository at: [doi.org/10.5061/dryad.gtht76ht1]. Sanger sequences of isolates are available on GenBank, details in SI Methods Table 3, accession numbers (PP576370-PP576373; PP554507-PP554509; PP564910-PP564911).
