# Supplementary material for: Symbiotic bacteria and fungi proliferate in diapause and may enhance overwintering survival in a solitary bee
Source: ISME J. 2024 May 20;18(1):wrae089. doi: 10.1093/ismejo/wrae089 (PMC11177884; doi:10.1093/ismejo/wrae089)
Supplement: Supplementary_Figures_Christensen_etal_2024_wrae089 [file supplementary_figures_christensen_etal_2024_wrae089.pdf]

Supplementary Figures:

Symbiotic bacteria and fungi proliferate in diapause and may enhance overwintering survival in a solitary bee

Shawn M. Christensen; Sriram Srinivas; Quinn S. McFrederick; Bryan N. Danforth; Stephen L. Buchmann; Rachel L. Vannette

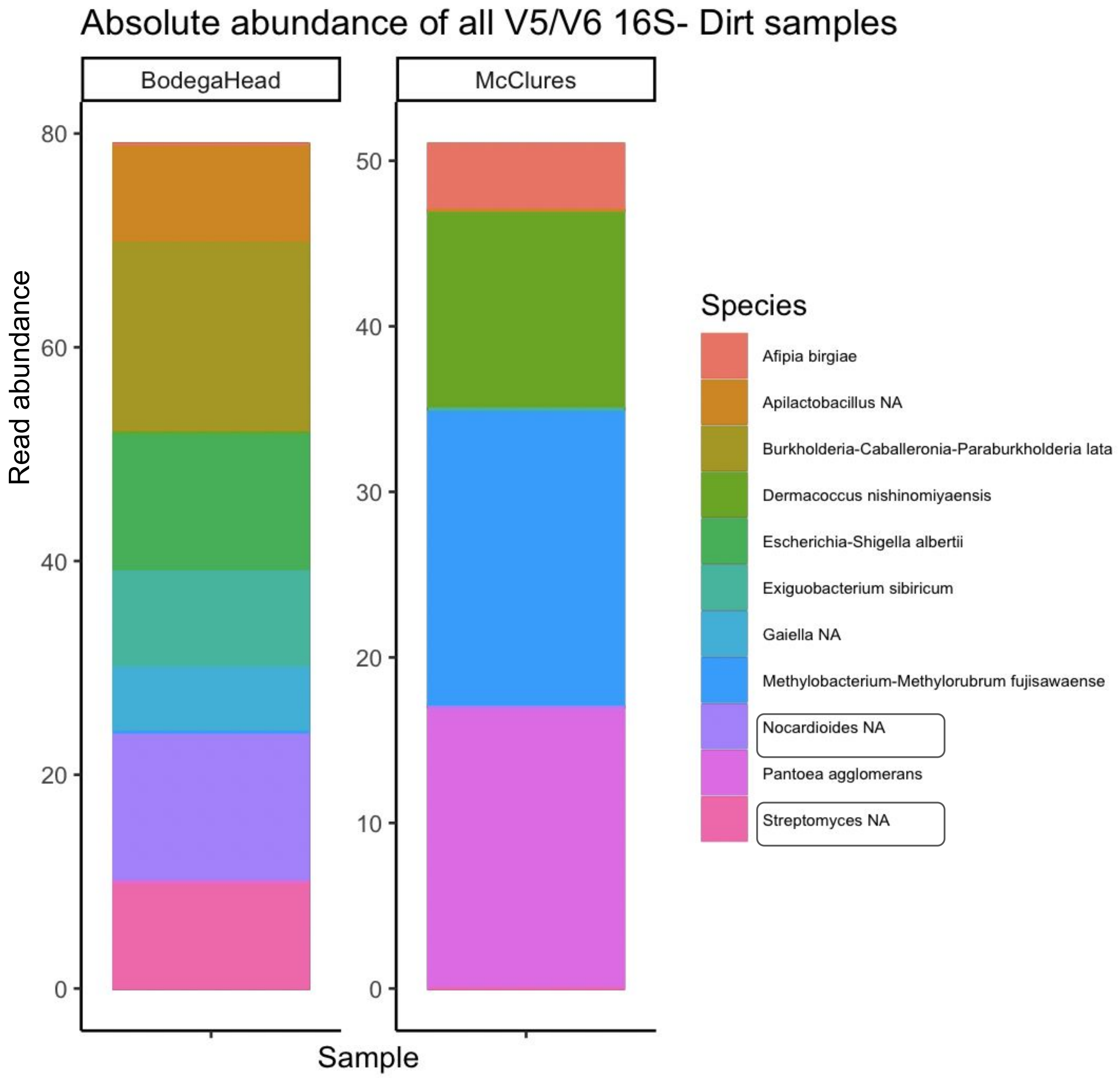

Fig. S1- Soil sample microbial composition. Soil samples were collected from each site, from ~2" beneath the surface, within the area of the nesting aggregation but not touching any nest. They were analyzed separately from the rest of the samples due to very low read count (note y axis), using a less stringent pipeline, and without using Decontam. *Nocardiodes* and *Streptomyces* (circled in key) were present in soil at Bodega Head but not found at McClure's. In separate analysis of fungal reads, no reads passed merge step: manual analysis showed there were 20 total reads in the McClure's soil sample, two of these reads were *M. spathulata* (BLAST), the rest were non-fungi.

Supplementary Figures:

Symbiotic bacteria and fungi proliferate in diapause and may enhance overwintering survival in a solitary bee

Christensen *et al* 2024

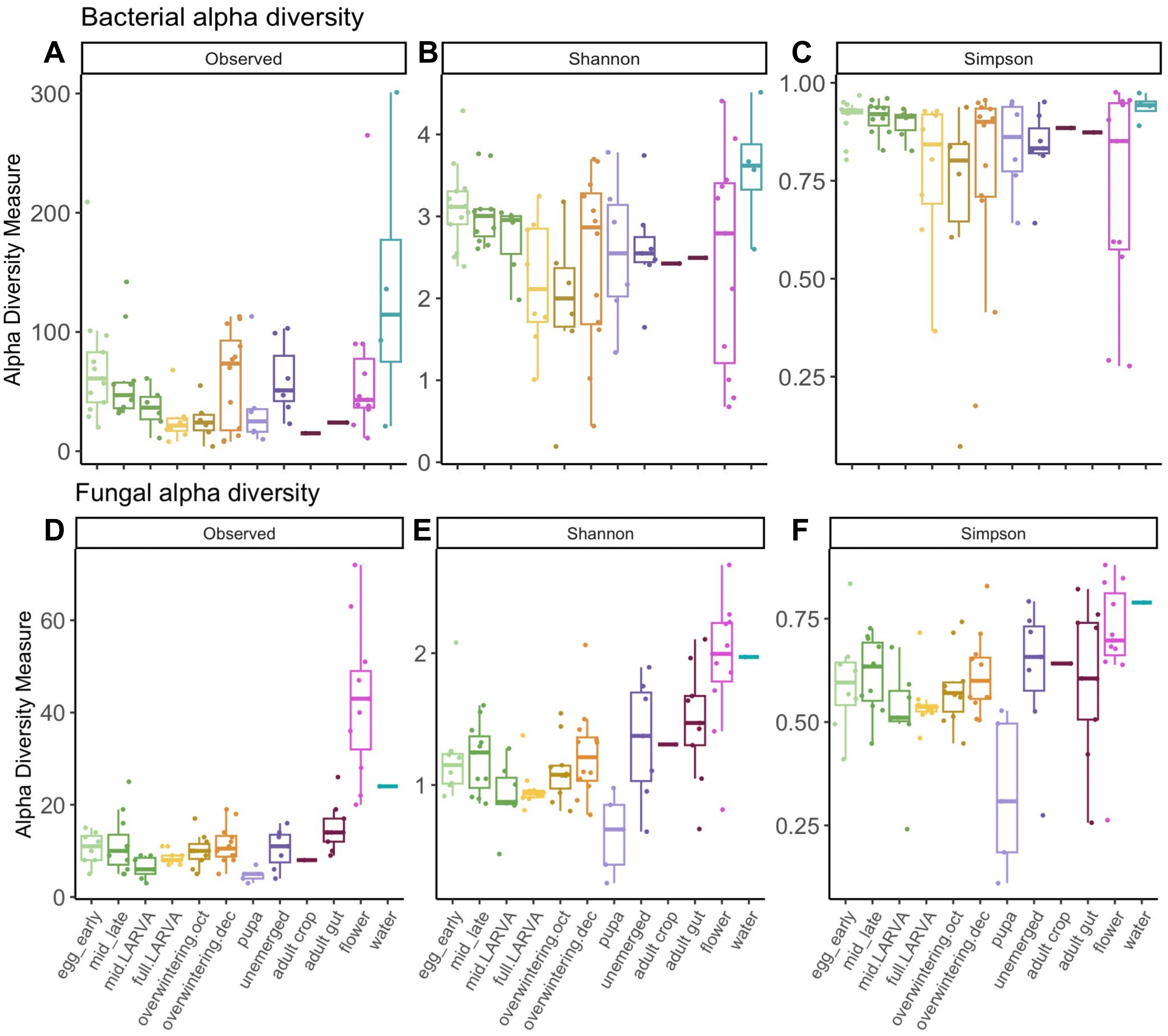

Fig. S2- Bacterial and fungal alpha diversity by developmental stage and sample type. Alpha diversity metrics (y axis) by sample type (x axis). No ASVs removed. **(A-C)** Bacterial alpha diversity: Observed alpha diversity sample type (x axis) is significant globally but no comparisons remain significant after *P* value correction. (Kruskal-Wallis χ^2= 22.838, df = 11, p= 0.01863). Shannon index does not differ globally by sample type (Kruskal-Wallis χ^2= 16.616, df = 11, *P* value > 0.05). Simpson diversity metric does not differ globally by sample type (Kruskal-Wallis $\chi 2$ = 15.295, df = 11, *P* value > 0.05). **(D-F)** Fungal alpha diversity varies significantly by sample type in all measurements (Observed: Kruskal-Wallis $\chi 2$= 51.8, df = 11, *P* value = 3e-7; Shannon: Kruskal-Wallis $\chi 2$= 39.4, df = 11, *P* value =4.5e-5; Simpson: Kruskal-Wallis $\chi 2$ = 26.9, df = 11, *P* value = 0.005).

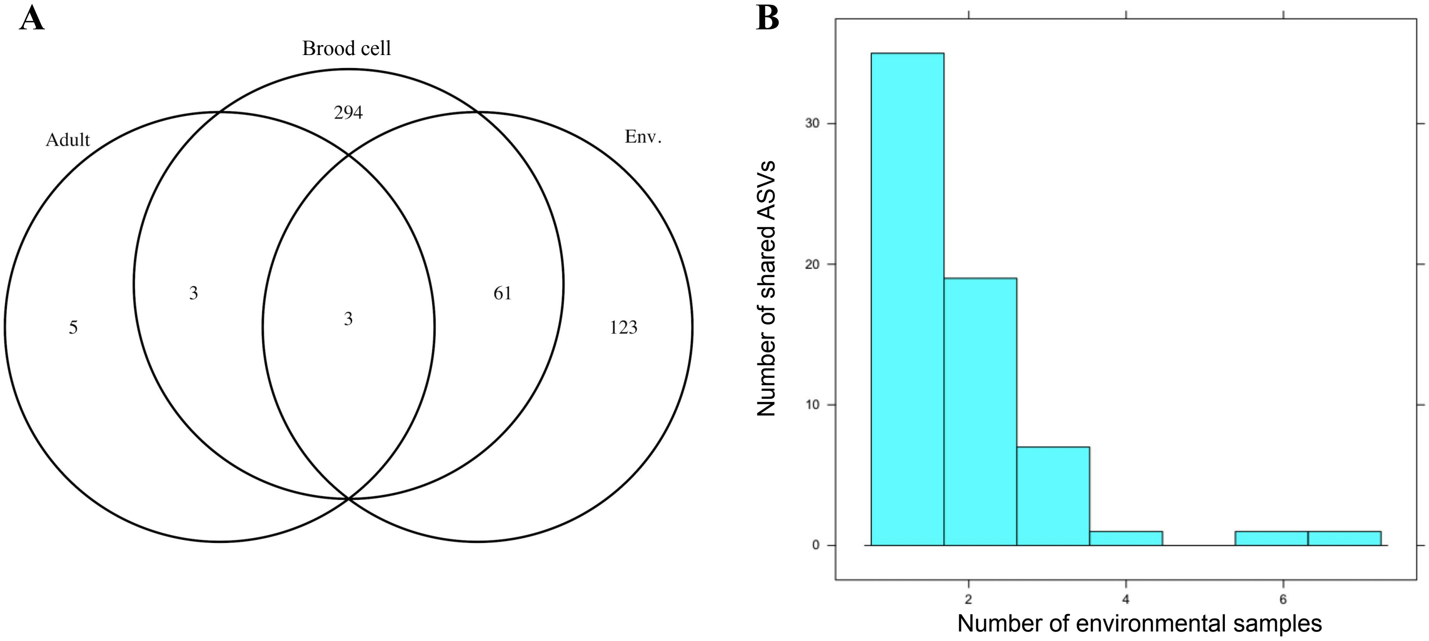

Fig. S3- Overlap of Actinobacterial ASVs with environmental ASVs does not fully explain acquisition. To assign ASV presence within a sample, we used a cutoff of 0.1% relative abundance, and filtered to only ASVs assigned to Actinobacteria.

A) Three ASVs: ASV_29, ASV_78, ASV_471 (Assigned as *Nocardioides*, *Mycobacterium*, and order: Frankiales, respectively) were found to occur in all sample groups (eg, adult, brood cell, environment). ASV_29 was found in 53.2% of all samples, ASV_78 was found in 23.9% of all samples, ASV_471 found in 4.3% of all samples. The mean relative abundances (in samples where the ASV was found) are ASV_29 mean= 0.017 SD= 0.014; ASV_78 mean = 0.015, SD= 0.015; ASV471 mean= 0.0013 SD=0.01. In total, 64 Actinobacteria ASVs overlap between brood cell and environmental samples, which represents less than one fifth (17.7%) of the Actinobacterial ASVs found in brood cell samples. Brood cell n=69, environment n=15, adult n=2.

B) Histogram of the 64 ASVs shared between brood and environmental samples, showing their representation in environmental samples. The vast majority (54 ASVs, 90%) of these ASVs were found in only one or two environmental samples (the first two bars on the left). The environmental samples contributing the most shared Actinobacterial ASVs were: Radish flower (15x flowers bulked) which contained 31 shared ASVs, and Sea Daisy (3x flowers bulked), which contained 27 shared ASVs.

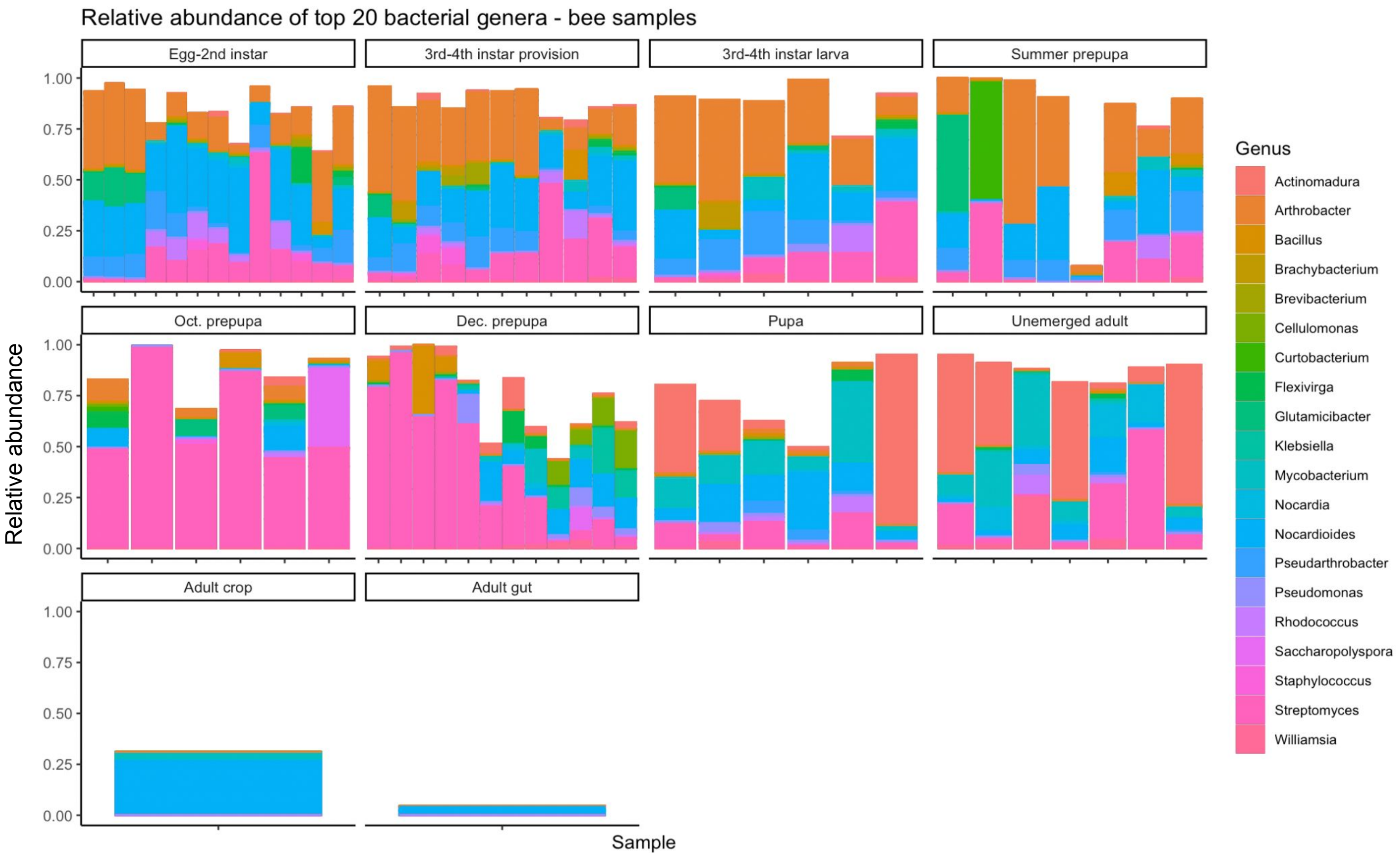

Fig. S4- Top 20 bacterial genera comprise the majority of reads in each stage, in varying abunances. Data subset to include only ASVs belonging to the 20 most abundant genera, and the white space indicates proportion of sample comprised by additional genera. ASVs are grouped and colored by genus, shown as relative abundance in sample. Each bar represents one sample, and samples are grouped by stage.

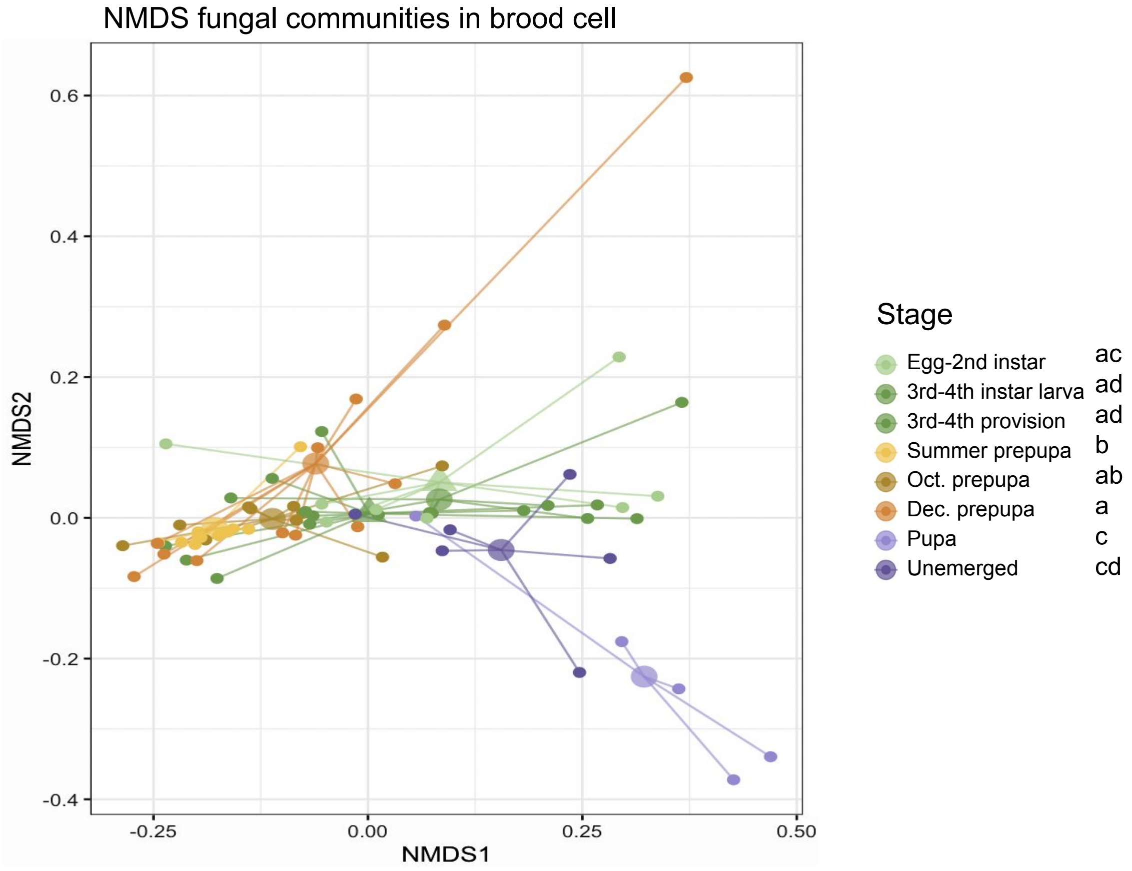

Fig. S5- Fungal communities shift with bee development. NMDS plot of Bray-Curtis distance, with color indicating stage of brood cell development. Larger semi-transparent dots indicate centroids, with lines from centroid to each point in the group. Triangular centroids indicate provision samples. Fungal- NMDS (stress=0.09) global PERMANOVA shows significant difference between stages (R^2=0.28, F=3.52, P value <0.001). Pairwise PERMANOVA of stages (P value <0.05, FDC corrected) indicated with lettering on figure key. N=71.

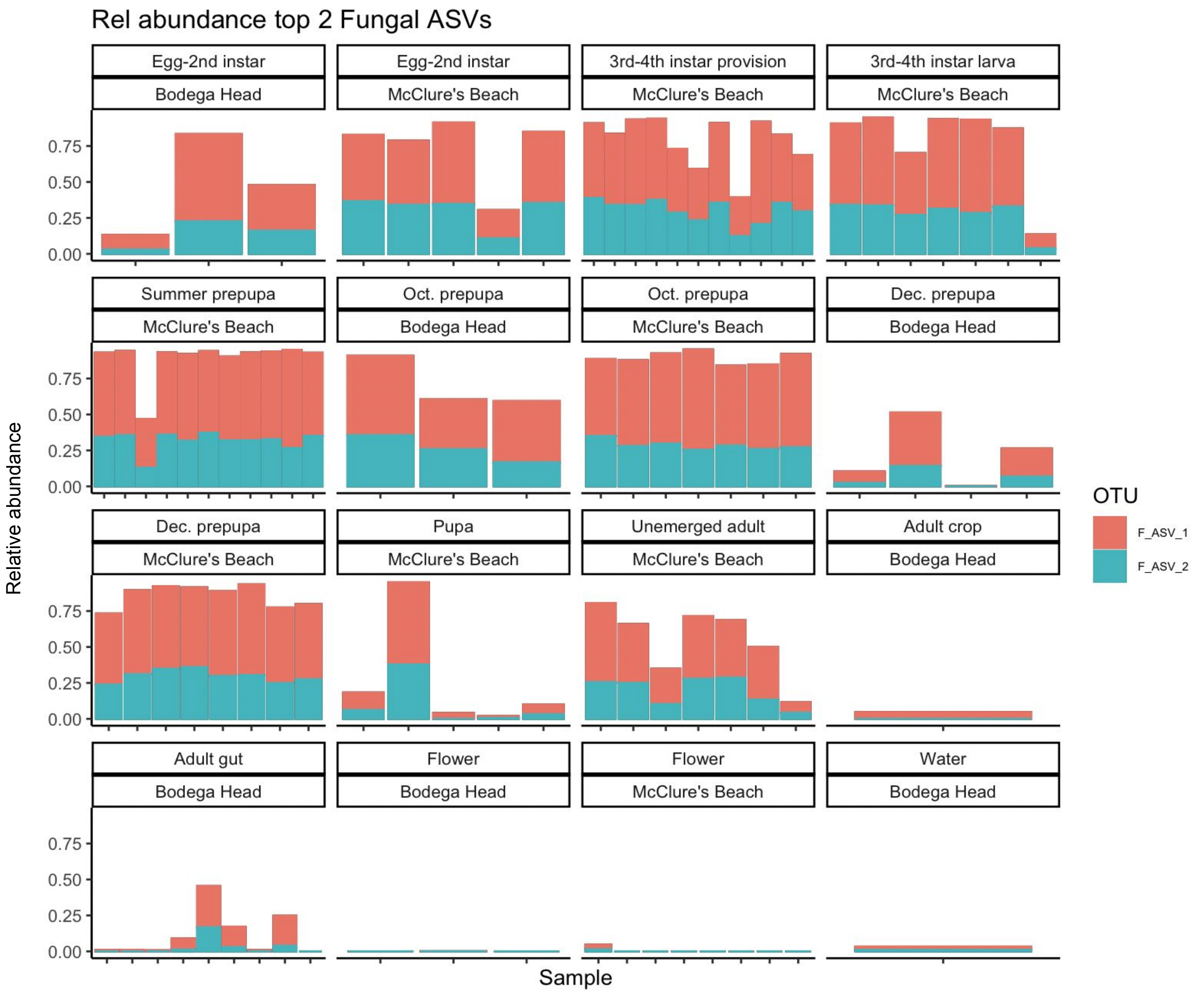

Fig. S6- Two ASVs of *Moniliella spathulata* comprise the majority of fungal reads in most brood cell samples and are found at both sites. Relative abundance data is shown, subset only to the top two ASVs in the fungal dataset, which are both assigned to *Moniliella spathulata*. Each bar represents one sample, samples are separated by stage or sample type and by collection site.

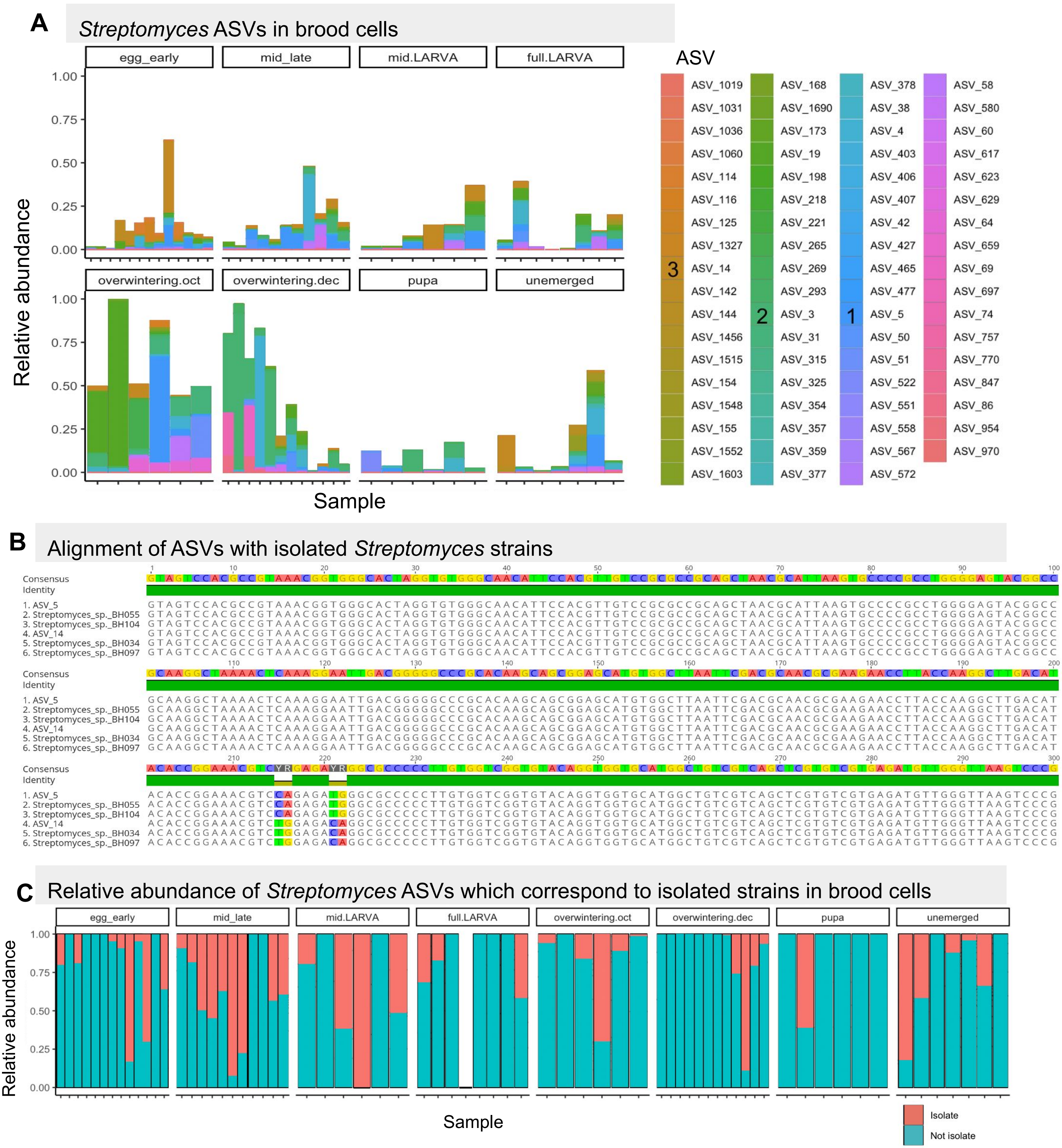

Fig. S7- ***Streptomyces*** **ASVs are variable in brood cells, but major ASVs are likely represented by isolated strains. A)** Relative abundance data is shown, subset to only show *Streptomyces* genus from the bacterial dataset. Each bar represents one sample, samples are separated by stage. Top three ASVs by total read abundance are labeled in the key (ASV_5, ASV_3, ASV_14, respectively). **B)** *Streptomyces* isolate 16S rRNA gene sequences match the sequenced region (V5/V6) of two abundant ASVs. Alignment created via MUSCLE v. 5.1. The most abundant *Streptomyces* ASV, ASV_5, matches exactly with isolates BH55 and BH104, while ASV_14, the third most abundant *Streptomyces* ASV, matches exactly with isolates BH34 and BH97. **C)** Relative abundance of ASVs 5 and 14 (combined, in red), which correspond to isolated *Streptomyces* strains ("isolate"), compared to other ASVs within *Streptomyces* genus that did not align with isolated strains ("Not isolate", in blue).

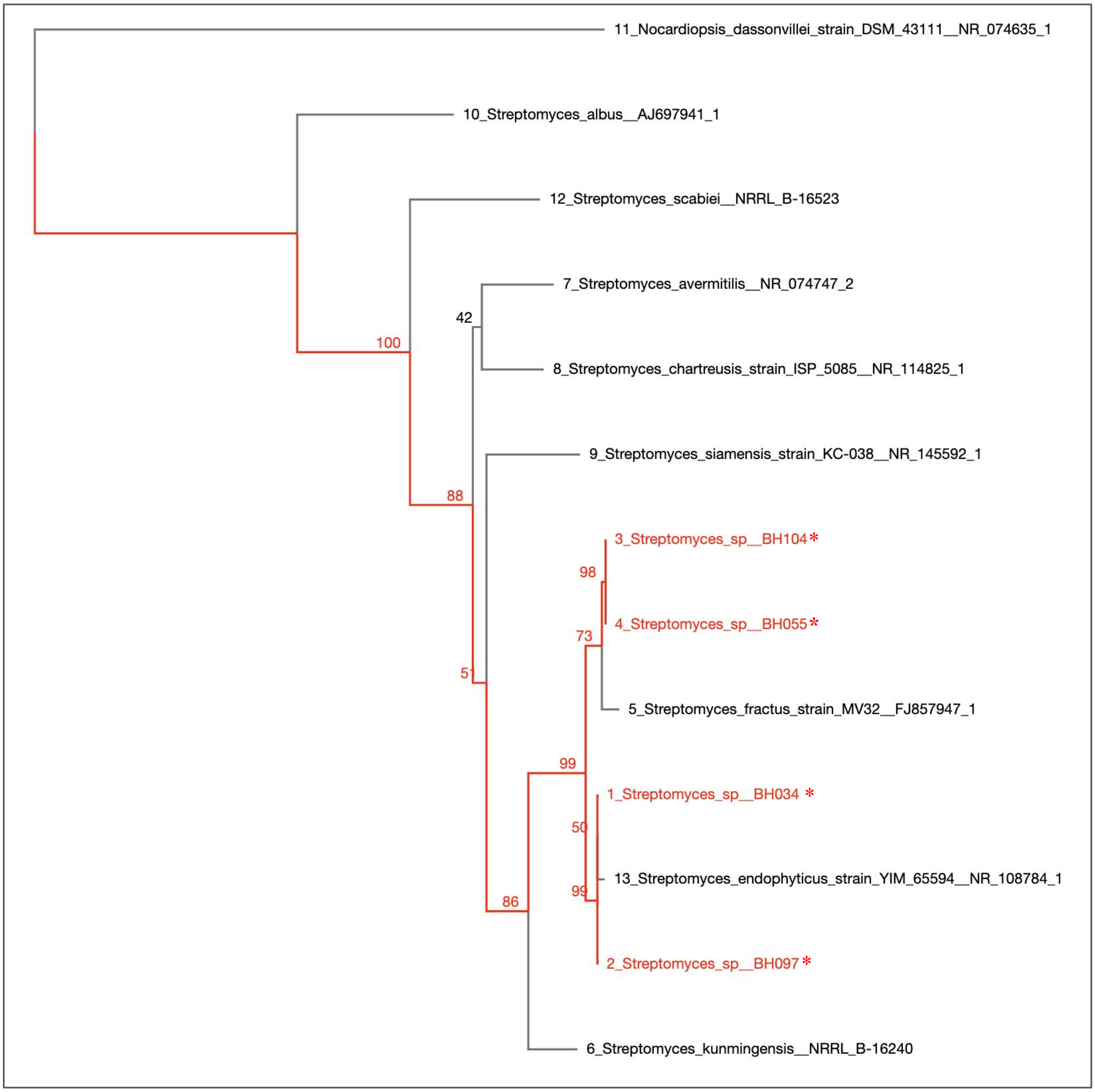

Fig. S8 - Phylogenetic tree showing the position of *Streptomyces* isolates from *A. bomboides* amongst closest phylogenetic neighbors. Alignment constructed with MAFFT using default parameters (method= L-INS-i), followed by tree construction using conserved sites (1203 sites, method= neighbor-joining, model= Jukes-Cantor, bootstrap resampling = 1000). Bootstrap support for each node is indicated as a percentage, calculated from 1,000 randomly re-sampled datasets. Isolates from this study were sequenced with Sanger using 27F/1492R primers, they are shown in red and are labeled with an asterisk (See SI Methods Table 3 for accession numbers). Other sequences were obtained from NCBI, accession numbers follow double underscore after isolate name.
