# Supplementary material for: Symbiotic bacteria and fungi proliferate in diapause and may enhance overwintering survival in a solitary bee
Source: ISME J. 2024 May 20;18(1):wrae089. doi: 10.1093/ismejo/wrae089 (PMC11177884; doi:10.1093/ismejo/wrae089)
Supplement: SI_Methods_wrae089 [file si_methods_wrae089.docx]

**Supplemental Information: Methods**

Symbiotic bacteria and fungi proliferate in diapause and may enhance overwintering survival in a solitary bee

Shawn M. Christensen; Sriram Srinivas; Quinn S. McFrederick; Bryan N. Danforth; Stephen L. Buchmann; Rachel L. Vannette

Sample collection and processing

Sites:

McClure’s Beach (Point Reyes National Seashore, CA, USA) is the larger nesting site, with roughly 2000-3000 nests estimated in early June of 2021 which would indicate around 1000-1500 or more active females given females are likely to have started a second nest by that time [1]. It is located in a WSW facing eroded bluff made of hard granitic sedimentary substrate (70% sand, 12.2% silt, 14.5% clay) and is ~150’ from the ocean. The Bodega Head nesting aggregation (Bodega Head Reserve, CA, USA) is smaller, estimated 250-500 nests; 100-250 active females (June 2021) and located on gently sloping sides and tops of eroded ditches. The substrate is finer and darker (76.5% sand, 9% silt, 14.1% clay). The site is also on the Pacific Ocean, though higher than McClure’s, ~50’ above sea level. The two sites are 9.8 miles apart, as the crow flies, separated by a 5-mile stretch of open water (Bodega Bay). Soil makeup determined by Cornell Soil Health Lab.

Weather and Climate:

Because of the largely Mediterranean climate, the nesting period is warm and quite dry (10.2/ 22.2°C average low/high; ~1.5” rain total from May-Sept), but winter is wetter and cooler (5.0/15.7°C average low/high; ~18.7” rain total from Nov-Feb) and there are on average 7.3 days where it drops to or below freezing (Nov-Feb). Data from Bear Valley Visitor’s center on Point Reyes via PRISM, averages for 2006-2015.

Collection:

Samples were collected from 2021-2023 at Point Reyes National Seashore (permit #: PORE-2020-SCI-0022) and Bodega Head (SCSP permit issued 2/24/2020). Adults were collected in June with a net while foraging or as they emerged from their nests in the early morning. To collect brood cell samples, small chunks of the cliff in the nesting area were separated using a soil knife and rock pick. These were then carefully dissected to separate brood cells, which are distinct and can be entirely removed from the surrounding soil matrix. Brood cells were then carefully opened from the top with sterilized tweezers or scoopulas (70% ethanol). Tweezers and/or scoopula were re-sterilized before being used to remove brood cell contents into sterile tubes and between brood cell samples. Egg-2^nd^ instar stage brood cells have a high proportion of nectar in provisions; in some cases, a pipette was used to transfer these provisions. Upon collection, samples were rated for how ‘clean’ the extraction of provision was (eg. some had more dirt fall in, or a larva was punctured by the tweezers and soil then stuck to it, etc). The developmental stage, location, and date of collection were also recorded. Developmental stages are described in Table 1. Tubes with collected samples were placed immediately into a cooler for transport back to the lab. Some were vortexed in a buffer (Phosphate Buffered Saline, PBS) and plated for bacterial and fungal isolation (Tryptic Soy Agar, TSA and Yeast Media, YM) or plated directly. The samples that were used for DNA extraction were moved directly from the cooler into a -80C freezer until sample processing.

| **Table 1** | | | | |
| --- | --- | --- | --- | --- |
| Stage | Approximate Phenology | Description, classification criteria | Samples  (collected, bacteria, fungi **) | Image |
| Egg-2^nd^ instar | Apr- Jul | Provision consists of majority nectar and is therefore very liquid. Egg or very small larvae may or may not have been found in the provision, and was not specifically separated or included. Egg through 2nd instar larvae. | N=13, 13, 8 | 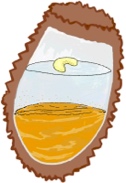 |
| 3^rd^-4^th^ instar | May- Jul | Provision: nectar has been mostly consumed, leaving pollen of play-dough consistency. | N=11, 11, 11 | 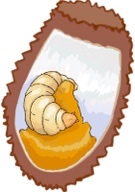 |
|  |  | Larvae: cream- colored and not yet full size, active and consuming provision. Larvae separated from provision for analysis. 3rd to 4th instar larvae. | N=10, 6, 7 |  |
| Summer Prepupae* | Jun-Aug | Prepupae: Have finished provision and consumed the cell lining. Color shifts to anywhere from pale yellow to bright orange at this stage. Defecation occurs, and prepupa enters diapause. | N=11, 8, 11 | 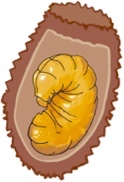 |
| October prepupae | Oct | Prepupae: In diapause, less turgid than late summer but otherwise appearance unchanged. | N=10, 6, 10 | 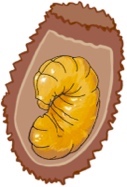 |
| December prepupae | Dec | Prepupae: In diapause, similar in size and appearance to prior two stages. | N=12, 12, 12 | 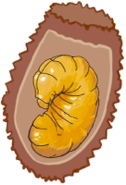 |
| Pupa | Mar- May | After diapause is broken, pupation occurs. No cocoon is spun. Collected pupae ranged in melanization from entirely pale yellow to near entirely melanized, extent was noted. | N=7, 6, 5 | 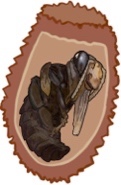 |
| Unemerged adult | Apr- May | Adults collected as they chewed out of their brood cells, or became active upon breaking open of brood cell. Hairs present, wings fully formed. | N=7, 7, 7 | 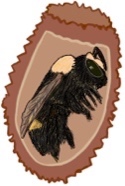 |
| Adult | Apr- Jul | Free-flying adult females collected while foraging or when leaving their nest after they were seen entering said nest. Adults were dissected, crops and guts were isolated for analysis | Guts:  N=10, 1, 9  Crops:  N=10, 1, 1 | 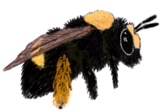 |
| * Bees remain prepupae from the time they consume the lining/defecate through diapause, thus the use of “Summer” to describe this initial prepupal stage.  **First number corresponds to total samples collected and submitted for sequencing, second corresponds to number of samples which passed filtering after 16S rRNA gene sequence analysis (bacteria), and third corresponds to number of samples which passed filtering in ITS region sequence analysis (fungi). | | | | |

Flowers, water, and soil were also collected from each location to better understand the environmental microbes that the bees encounter (Table 2). *Anthophora bomboides* females use fresh water to soften the dirt that they nest in, and often do so in aggregate at a specific location on the spring’s edge. This water was collected into sterile collection tubes from each site at the section that was actively in use by the aggregation at the time of collection. Flowers of each species were collected in June, when adults are foraging, and bulked upon collection into sterile tubes. Nesting substrate, here called “soil”, was collected from within the aggregate nesting area at each site, from within 2” of the cliff surface, several inches from any nests, but within the nesting area. It was collected by chiseling away the exposed surface and then scraping underlying material into a collection tube with a sterile soil knife.

| **Table 2** | | | | |  |
| --- | --- | --- | --- | --- | --- |
| Type | Species | Description | Samples  (collected, bacteria, fungi **) | | |
| Flowers | *Lupinus arboreus*  Sims | Yellow bush lupine: preferred pollen source One from each site, each sample containing 5-7 bulked flowers | N=2 | N= 12, 11, 11 | |
|  | *Raphanus sativus* L. | Wild radish: preferred nectar source  Two from McClure’s, one from Bodega site, each sample containing ~10-15 flowers | N=3 |  |  |
|  | *Eschscholzia californica* Cham. | California poppy: pollen source  McClure’s, 2 bulked flowers | N=1 |  |  |
|  | *Carpobrotus edulis* L. | Ice plant: nectar source  Bodega, 1 flower | N=1 |  |  |
|  | *Cacklile maritima* Scop. | Sea rocket: nectar source  McClure’s, 10 bulked flowers | N=1 |  |  |
|  | *Grindelia stricta* DC. | Gum plant: nectar and/or pollen source  McClure’s, 3 bulked flowers | N=2 |  |  |
|  | *Erigeron glaucus*  Ker Gawl. | Seaside daisy: nectar and/or pollen source  McClure’s, 3 bulked flowers | N=2 |  |  |
| Soil | NA | Nesting material from the surrounding nest site, but not touching any nest, ~2” into nesting surface. (one per site*) | N=2, 0, 0 | | |
| Water | NA | Water from the site where adult females collect water for nest construction.  Two from each site | N=4, 4, 4 | | |
| Blanks | NA | No sample added, to ensure sterility of extraction kit. Blanks were spaced out across rounds of extractions and used in DECONTAM. | N=4, 0, 0 | | |
| * Soil samples were collected from both sites, both failed to pass through the initial DADA2 pipeline due to very low reads, which was confirmed with qPCR (Fig. 4), so a second, more relaxed DADA2 pipeline was run for the soil samples to determine general composition, see Fig. S1.  **First number corresponds to total samples collected and submitted for sequencing, second corresponds to number of samples which passed filtering after 16S rRNA gene sequence analysis (bacteria), and third corresponds to number of samples which passed filtering in ITS region sequence analysis (fungi). | | | | | |

#### Sample pre-processing:

For collected brood cell samples that had a visible larva and remaining pollen provision (3rd-4^th^ instar), the larvae were separated from the provision and proceeded as two separate samples. Larvae and prepupae were all rinsed gently by pipetting in and removing sterile PBS (2x) before DNA extraction to remove dirt that was introduced in the opening of the brood cells or clinging provision material. For all bee samples, the cleanest samples of each stage, as rated upon collection, were used. Free-flying adult females were dissected, separating the crop and gut as two separate samples per bee. Water samples were centrifuged at 13k for 3 minutes to concentrate the sample for DNA extraction. Top water was removed, and the remaining 100ul was used to resuspend the pelleted solids for extraction. Flower samples were immersed in PBS and vortexed at max speed for 60 seconds to dislodge microbes into the PBS, then whole flower material was removed, and the tubes were centrifuged at 13k for 3 minutes. Top PBS was removed, and the remaining 100ul was used to resuspend the pelleted solids.

Microbe isolation and identification

Brood cells, flowers, and water samples that were not used for DNA extraction were plated to isolate bacteria and fungi. These were plated either directly or after suspension in PBS onto both TSA +cycloheximide and YM+ chloramphenicol plates. Once colonies grew (1-5 days), representative colonies (based on morphology) were picked onto separate isolation plates of the same type and allowed to grow. Pure strains were then named based on isolation site (BH= Bodega Head, PR = Point Reyes (McClures); and saved as glycerol stocks in the -80. A subset of these were then identified via colony PCR (27F/1492R for bacteria, ITS1F/2 or NLR1/4 for fungi) followed by Sanger sequencing at the UC Davis DNA sequencing core, and NCBI BLAST.

DNA Extraction

All samples were added whole to DNA extraction, following preprocessing. Extraction for all samples was done per manufacturer’s instructions with the DNeasy PowerSoil Pro kit. Four blanks were included in DNA extractions (Table 1). Extracted DNA was stored in the included extraction buffer at -80^o^C for amplicon sequencing and qPCR.

Amplicon sequencing

Amplicon sequencing of extracted DNA was done to assess bacterial and fungal community composition using the 16S rRNA (V5/6) gene and ITS region at the Integrated Microbiome Resource (IMR) at Dalhousie University in Halifax, Nova Scotia. Phusion Plus high-fidelity polymerase was used with fusion primers, which include the sequences below with Illumina adaptors + indices for multiplexing; sequencing was then performed on Illumina MiSeq [2, 3]. Samples were de-multiplexed at IMR. For bacteria, primers 799F/1115R amplifying V5/V6 region of the 16S gene were used to limit mitochondria and chloroplast amplification (799F= 5'-AACMGGATTAGATACCCKG-3'/ 1115R= 5'-AGGGTTGCGCTCGTTG-3')[4]. These primers amplify a ~300bp length target sequence. For fungi, primers ITS1F/ITS2 were used (ITS1F= 5'-CTTGGTCATTTAGAGGAAGTAA-3'/ ITS2=5'-GCTGCGTTCTTCATCGATGC-3'). These primers amplify the variable length ITS1/2 region.

### Amplicon data analysis:

Sequences were analyzed in R (4.1.1)[5] with primarily the DADA2 package (1.22.0) [6], phyloseq (1.38.0) [7], vegan (2.6.4)[8], microbiome (1.23.1)[9] and ggplot2 (3.4.2)[10]. See code for further details.

#### Bacteria:

Reads were filtered and trimmed with the following parameters (others were default): maximum expected error was set to 2 for forward reads and 5 for reverse reads (to account for lower quality of reverse reads), reads were truncated at 280 and 220, respectively, to discard bases with quality scores <~30). Primers were removed by trimming the respective primer length. Error rates, dereplication, denoising, merging, and chimera removal were done with default parameters; see supplemental code (Bacteria, code1) and data (‘16S_track_reads’). ASVs were inferred via DADA2 (1.22.0) and then taxonomy was assigned using the Silva ​​138.1 N99 database for bacteria [11]. Mitochondria and chloroplast assigned reads were removed. Decontam package (1.14.0)[12] was used to identify and remove potential contaminants by comparing blanks to samples; 5 were found and removed at the threshold parameter of 0.5. Samples with less than 300 reads were then removed from further analysis, leaving N=86 samples; see supplemental code (Bacteria, code2). Samples lost (37) were: 4/4 blanks, 9/10 adult crop, 9/10 adult gut, 2/2 dirt (analyzed independently), 1/1 ice plant flower, 4/10 3rd-4^th^ instar larvae, 3/11 Summer prepupae, 4/10 Oct. prepupae, 1/7 pupae.

#### Fungi:

Primers were removed using Cutadapt [13]. Reads were filtered and trimmed using default parameters, aside from the length minimum, which was set to 70 to remove extremely short reads. Error rates, dereplication, denoising, merging, and chimera removal were done with default parameters; see supplemental code (Fungi, code1) and data (‘ITS_track_reads’). ASVs were inferred via DADA2 (1.22.0). We assigned fungal reads with the UNITE 9.0 general release dynamic database (29.11.2022)[14]. Non-fungal assigned reads were then removed, and Decontam package (1.14.0)[12] was used to remove potential contaminants by comparing blanks to samples; two were found and removed at threshold=0.5. Samples with fewer than 300 reads were then removed from further analysis, leaving N=93 samples. Samples lost (26) were: 4/4 blanks, 5/13 1st-2nd instar, 3/10 3rd-4th instar larvae, 1/1 dirt sample (analyzed independently, Fig. S1), 9/10 adult crops, 1/10 adult guts, 1/1 gum flower, 2/7 pupae.

Community differences:

To evaluate the compositional differences in microbial communities based on occurrence inside or outside of the brood cell, as well as for stage specific community separation, amplicon sequence data was used to create separate Bray-Curtis (BC) dissimilarity matrices for both bacteria and fungi. These were ordinated with NMDS (Fig. 2C,D). PERMANOVA was run based on BC distances to determine differences in community composition by sample type (in brood cell, out of brood cell, water, flowers) for both bacteria and fungi.

Relative abundance of Actinobacteria and *Moniliella* inside vs outside of brood cell:

Data was subset to ASVs assigned to the Actinobacterial class (or *Moniliella spathulata* species). Samples were grouped based on whether they occur inside or outside of the brood cell (egg through unemerged adult: in brood cell; adult, flower and water samples: outside of brood cell). The total relative abundance of Actinobacteria (or *Moniliella spathulata*) assigned ASVs were compared between the groups with the Base R ‘stats’ package (4.1.1) [5] ‘kruskal.test’ function.

Defining the core microbiome:

Detection of a core microbiome occurs using prevalence and abundance (detection) thresholds, and these can vary widely by study system, environment, and goals of the analysis [15, 16]. Therefore, we used the ‘microbiome’ package “plot_core” function to visualize a wide range of prevalence (0 to 100%) and abundance (0.01% to 20%) thresholds for both bacteria and fungi in the style of a heatmap (Fig. 3) for clarity, and to allow for nuance in interpretation of what may be considered core taxa.

Abundance of *Streptomyces* by stage:

Data was subset to ASVs assigned to the genus *Streptomyces*, and samples were grouped based on season, with egg- summer prepupa as ‘summer’, October and December prepupa as ‘overwintering’, and pupa- unemerged adults as ‘spring’. Differences in relative abundance of *Streptomyces* of these groups was evaluated with the Base R ‘stats’ package (4.1.1) [5] ‘kruskal.test’ function followed by the ‘FSA’ package (0.9.4)[17] ‘dunnTest’ function with Bonferroni p-value correction [18]. To determine ‘actual’ abundance, we combined the qPCR data with the amplicon data by multiplying the total bacterial copy number by the proportion assigned to *Streptomyces* in each sample*.*

qPCR- Microbial copy number

#### Bacteria:

Bacterial copy number was quantified with standard DNA intercalating dye (SYBR) based qPCR. The same extracted samples that were sent for amplicon sequencing were run through this procedure. Identical primers (799F= 5'-AACMGGATTAGATACCCKG-3' /1115R= 5'-AGGGTTGCGCTCGTTG-3') were used so that compositional and quantification could be directly compared and merged. A 1:10 dilution of extracted DNA was determined after dilution testing was done with a representative subset of samples; 1:10 dilution gave in-range Cq values. Master mix, per reaction, was composed of 5ul SSO Advanced Universal SYBR Supermix (Catalog# 1725271), 0.3ul of each primer (10uM), 3.4ul Molecular grade water, and 1ul of extracted DNA (diluted 1:10 in Molecular grade water). Reactions were performed in triplicate for each sample, and arranged semi-randomly across plates to avoid possible correlations of plate and developmental stage. Blanks and standards were included in each plate, and a Cq cutoff for blanks was established at 31.

To translate Cq values to copy number, we purchased a plasmid containing the relevant sequence from *Nocardiodes luteus (*ASV_5) from Eurofins at a known concentration. This was diluted in 10-fold steps; the dilution steps of 1.17E+6 through 0.117 molecules/ul, plus a blank, were used to create a standard curve, which had an *R*^2^= 0.983. The equation of this line (see code) was used to convert Cq values to log (copy number), and edited to account for dilution.

Using amplicon data, we also adjusted the final qPCR copy number to remove the proportion of reads in each sample that had been assigned to mitochondria (no chloroplast reads were assigned). Flower and water samples had to be concentrated to ensure sufficient DNA for sequencing during pre-processing and thus bacteria in these samples could not be reliably quantified with qPCR.

#### Fungi:

Fungal copy number was quantified with probe-based (Taq-Man®) qPCR using a previously established system, FungiQuant [19]. Because the ITS region is highly variable in length, we did not use the same approach as above, as SYBR will intercalate throughout the length of an amplicon, potentially resulting in higher fluorescence readings for samples with a greater proportion of longer ITS amplicons. For these reasons, the fungal quantification was done with FungiQuant, using the 18S rRNA gene primers FungiQuant-F= 5′-GGRAAACTCACCAGGTCCAG-3′ and FungiQuant-R = 5′-GSWCTATCCCCAKCACGA-3′, along with the fluorescent probe FungiQuant-Prb = (6FAM) 5′-TGGTGCATGGCCGTT-3′ (MGBNFQ). As with bacteria, dilution testing of samples was done to bring Cq values into the optimal range, and a 1:20 dilution was picked. Master mix, per reaction, was composed of 5ul PCR Biosystems qPCRBIO Probe Mix (No-ROX) (Catalog# 17-512B), 0.3ul of each primer (10uM), 0.3ul fluorescent probe (10uM), 3.1ul molecular grade water, and 1ul of extracted DNA (diluted 1:20 in molecular grade water). Reactions were performed in triplicate for each sample, and arranged semi-randomly across plates to avoid possible correlations of plate and developmental stage. Blanks and standards were included in each plate.

To translate Cq values to copy number, we purchased a plasmid containing the relevant sequence from *Moniliella oedocephalis* on NCBI (#NG_062174) from Eurofins at a known concentration. This was diluted in 10-fold steps and the dilutions steps of 1.28E+6 through 0.128 molecules/ul, plus a blank, were used to create a standard curve, which had an *R*^2^= 0.983. The equation of this line (see code) was used to convert Cq values to log(copy number), and edited to account for dilution.

Statistical Analysis:

To evaluate differences in copy number between stages for both bacteria and fungi, we used the Base R ‘stats’ package (4.1.1) [5] ‘kruskal.test’ function followed by the ‘FSA’ package (0.9.4)[17] ‘dunnTest’ function with BH p-value correction [20].

### Inhibition

Strain isolation and identification:

Strains of *Streptomyces* were isolated by plating brood cell contents on Tryptic Soy Agar (TSA) with added cycloheximide. Colonies were picked by hand and replated on TSA until isolated, then glycerol stocks were created. *Streptomyces* isolates were identified to closest taxa based on Sanger sequencing of the 16S rRNA gene (primers 27F/1492R) using NCBI BLAST (Table 3) [21]. To ensure that the isolates were representative of the diversity of *Streptomyces* in the brood cells (Fig. S7A), we aligned the 16S rRNA gene sequences from the isolates with the 300bp amplified region from our community ASV sequencing data using MUSCLE and found that the isolates likely represent two of the three most abundant *Streptomyces* ASVs (Fig. S7B, C) [22]. Additionally, the 16S rRNA gene sequences were aligned with those of known *Streptomyces* from NCBI using MAFFT with default parameters (method= L-INS-i) and a phylogenetic tree was constructed using conserved sites (1386 sites, method= neighbor-joining, model= Jukes-Cantor, bootstrap resampling = 1000) (Fig. S8) [23].

*Thelonectria* was isolated in much the same way, but from an infected *A. bomboides* pupa that had developed external filamentous fungal growth. *Moniliella spathulata* was isolated from a 1^st^ instar *A. bomboides* provision (though it also occurred in nearly every plated brood cell sample, and all identified to same BLAST ID). *Ascosphaera apis* and *Aspergillus flavus* were isolated from infected *Bombus impatiens* larvae previously in the Vannette lab. All fungal isolation occurred with Yeast Media Agar with added chloramphenicol. Identification was based on Sanger sequencing of the ITS or 18S rRNA D1/D2 region (primers ITS1F/ITS2 for *Thelonectria;* NL1/NL4 for *Ascosphaera apis*; ITS86F/ITS4 *Aspergillus flavus,* NL1/NL4 and ITS1/ITS4 for *Moniliella spathulata*) followed by NCBI BLAST [21] (Table 3).

| **Table 3** | | | |
| --- | --- | --- | --- |
| *Strain* | *BLAST ID* | *Isolation source* | *Accession numbers* |
| BH34 | *Streptomyces endophyticus strain YIM 65594* | 1^st^ instar *A. bomboides* provision | 27F/1492R (PP576370) |
| BH55 | *Streptomyces endophyticus strain YIM 65594* | Egg stage *A. bomboides* provision | 27F/1492R (PP576373) |
| BH97 | *Streptomyces endophyticus strain YIM 65594* | October *A. bomboides* prepupa | 27F/1492R (PP576371) |
| BH104 | *Streptomyces endophyticus strain YIM 65594* | October *A. bomboides* prepupa | 27F/1492R (PP576372) |
| FFP4 | *Thelonectria sp.* strain OTU1563 | *A. bomboides* pupa, infected | ITS1F/ITS2 (PP554508) |
| AA1 | *Ascosphaera apis* | *Bombus impatiens* larva | NL1/NL4 (PP564911) |
| BAIF1 | *Aspergillus flavus* | *Bombus impatiens* larva | ITS86F/ITS4 (PP554507) |
| BH004 | *Moniliella spathulata* | 1^st^ instar *A. bomboides* provision | ITS1F/ITS2 (PP554509) |
|  |  |  | NL1/NL4 (PP564910) |

Trials:

In order to ensure that both the bacteria and fungi could grow on the same media, for all inhibition trials we used TSA without any antimicrobials. For consistency, we used a template to mark the underside of all of the plates, it included in the very center a cross “+” then two parallel lines, 30mm long and each 20mm from the center point. These served as guides for inoculations. *Streptomyces* strains were inoculated with 1ul hoops from stock plates of the TSA without antifungals onto the 30mm parallel lines. Five replicate plates were made for each comparison (25 per trial, including 5 control plates). These were allowed to grow for 10 days, then, from stock plates of each fungus (also TSA, no antimicrobials), plugs were inserted into the center “+” of each plate. Care was taken to ensure that plugs were all taken from just inside the leading edge of the fungal hyphae on the stock plates. These were allowed to grow for seven days. Measurements were taken on the backs of the plate and measured the distance from the leading edge of the growing fungi to the center “+”, directly perpendicular to the parallel lines, on both sides. A tabletop light pad was used for imaging to qualitatively assess the density of the fungal hyphae, ensuring even back-lighting for the plates.

Analysis:

Radius measurements (two per plate, each side of the ‘+’) were averaged for each replicate plate. Kruskal-Wallis was run with the Base R ‘stats’ (4.1.1) package ‘kruskal.wallis’ function as radius by inhibition treatment. This was followed with multiple comparisons with the ‘FSA’ package (0.9.4)[17] ‘dunnTest’ function, but as we were only interested in comparisons to the negative control, we then subset to those four comparisons. P value correction done with ‘stats’ package ‘p.adjust’ function using a Bonferroni correction [18].

Sugar and Sugar Alcohols

Preparation:

Samples of whole larvae, prepupae and pupae, as well as one pollen provision from a 4^th^ instar larva were extracted for sugar and sugar alcohol analysis. Whole samples were placed in tubes with metal beads and 1mL of 100% ethanol and run on a bead beater for 8 minutes at full speed with 20s breaks every minute. These were then centrifuged for 30 seconds at 10k rcf. For each sample, the top 700ul of ethanol was moved to a new tube, 700ul 100% hexane was added, and then vortexed for 30 seconds. To this, 100ul MilliQ water was added, and vortexed for another 30 seconds. Once hexane had separated from the aqueous phase, it was removed (800ul). The remaining 1mL of aqueous phase was centrifuged for 2 minutes at 16k rcf, and the bottom 500ul was filtered through a 0.2 micron syringe filter and placed in a new tube in a lyophilizer for 6 hours, without heat. The dried samples were kept in a -20C freezer until analysis, at which time they were re-suspended in 300ul 1:1 water: acetonitrile. Standards of erythritol, sorbitol, fructose, glucose, sucrose, xylose and maltose were made at 0.5 mg/mL, standards of glycerol and trehalose were made at 5mg/mL and 1mg/mL respectively, all in 1:1 water: acetonitrile.

Separation:

Separation of sugars was performed on Thermo UltiMate 3000 HPLC system according to the Waters Application Note: WA60110, except for the following: column was Phenomenex Luna Omega 3um SUGAR (50x2.1mm, Part#: 00B-4775-AN), and flow rate was 0.2mL/min; detection was by CAD (Corona Veo; Dionex). Each sample was run twice, standards were run 2-5 times. Analysis of peaks was performed with Thermo Fisher Chromeleon software. Peak identities were assigned based on retention times of standards, and unassigned peaks were then named by their retention times. Peak area was calculated by the software and this data was exported for analysis.

Analysis:

To identify differences in sample groups based on SSA profiles we used Principal Components Analysis (PCA) ‘stats’ package, Base R [5]. Data was first normalized by Hellinger transformation. The ‘factoextra’ package was used to plot PCA and biplot of components. After calculation of Bray-Curtis distance matrix, PERMANOVA (‘vegan’ package; [8] and pairwise PERMANOVA (package ‘RVAideMemoire’ 0.9.83) were used to determine differences in composition of SSA by sample group, p-value correction by FDR [20].

References:

1. Brooks R. Biology of Anthophora bomboides standfordiana Cockerell. Systematics and Bionomics of Anthophora: The Bomboides Group and Species Groups of the New World. 1983. University of California Publications, pp 2–25.

2. Comeau AM, Douglas GM, Langille MGI. Microbiome helper: A custom and streamlined workflow for microbiome research. mSystems 2017; **2**: 10.1128/msystems.00127-16.

3. Comeau AM, Kwawukume A. Preparing multiplexed 16S/18S/ITS amplicons for the Illumina MiSeq. Protocols.io 2023.

4. Chelius MK, Triplett EW. The diversity of archaea and bacteria in association with the roots of Zea mays L. Microb Ecol 2001; **41**: 252–263.

5. R Core Team. R: a language and environment for statistical computing. 2020. R Foundation for Statistical Computing, Vienna, Austria.

6. Callahan BJ, McMurdie PJ, Rosen MJ, Han AW, Johnson AJA, Holmes SP. DADA2: High-resolution sample inference from Illumina amplicon data. Nat Methods 2016; **13**: 581–583.

7. McMurdie PJ, Holmes S. Phyloseq: An R package for reproducible interactive analysis and graphics of microbiome census data. PLOS ONE 2013; **8**: e61217.

8. Dixon P. VEGAN, a package of R functions for community ecology. J Veg Sci 2003; **14**: 927–930.

9. Lahti L, Shetty S. Tools for microbiome analysis in R. 2017.

10. Wickham H. ggplot2: Elegant Graphics for Data Analysis. 2016. Springer-Verlag New York, NY.

11. Quast C, Pruesse E, Yilmaz P, Gerken J, Schweer T, Yarza P, et al. The SILVA ribosomal RNA gene database project: improved data processing and web-based tools. Nucleic Acids Res 2013; **41**: D590–D596.

12. Callahan B, Davis NM, Ernst FGM. decontam: Identify contaminants in marker-gene and metagenomics sequencing data. 2023. Bioconductor version: Release (3.17).

13. Martin M. Cutadapt removes adapter sequences from high-throughput sequencing reads. EMBnet.journal 2011; **17**: 10–12.

14. Nilsson RH, Larsson K-H, Taylor AFS, Bengtsson-Palme J, Jeppesen TS, Schigel D, et al. The UNITE database for molecular identification of fungi: handling dark taxa and parallel taxonomic classifications. Nucleic Acids Res 2019; **47**: D259–D264.

15. Shade A, Handelsman J. Beyond the Venn diagram: the hunt for a core microbiome. Environ Microbiol 2012; **14**: 4–12.

16. Neu AT, Allen EE, Roy K. Defining and quantifying the core microbiome: Challenges and prospects. Proc Natl Acad Sci 2021; **118**: e2104429118.

17. Ogle DH, Doll JC, Wheeler AP. FSA: Simple fisheries stock assessment methods. 2023.

18. Bonferroni CE. Teoria statistica delle classi e calcolo delle probabilità. 1936. Seeber.

19. Liu CM, Kachur S, Dwan MG, Abraham AG, Aziz M, Hsueh P-R, et al. FungiQuant: A broad-coverage fungal quantitative real-time PCR assay. BMC Microbiol 2012; **12**: 255.

20. Benjamini Y, Hochberg Y. Controlling the false discovery rate: A practical and powerful approach to multiple testing. J R Stat Soc Ser B Methodol 1995; **57**: 289–300.

21. Altschul SF, Gish W, Miller W, Myers EW, Lipman DJ. Basic local alignment search tool. J Mol Biol 1990; **215**: 403–410.

22. Edgar RC. MUSCLE: multiple sequence alignment with high accuracy and high throughput. Nucleic Acids Res 2004; **32**: 1792–1797.

23. Katoh K, Rozewicki J, Yamada KD. MAFFT online service: multiple sequence alignment, interactive sequence choice and visualization. Brief Bioinform 2019; **20**: 1160–1166.
